# Haplotype-resolved genomes provide insights into structural variation and gene content in Angus and Brahman cattle

**DOI:** 10.1038/s41467-020-15848-y

**Published:** 2020-04-29

**Authors:** Wai Yee Low, Rick Tearle, Ruijie Liu, Sergey Koren, Arang Rhie, Derek M. Bickhart, Benjamin D. Rosen, Zev N. Kronenberg, Sarah B. Kingan, Elizabeth Tseng, Françoise Thibaud-Nissen, Fergal J. Martin, Konstantinos Billis, Jay Ghurye, Alex R. Hastie, Joyce Lee, Andy W. C. Pang, Michael P. Heaton, Adam M. Phillippy, Stefan Hiendleder, Timothy P. L. Smith, John L. Williams

**Affiliations:** 1 The Davies Research Centre, School of Animal and Veterinary Sciences, University of Adelaide, Roseworthy, SA 5371 Australia; 2 Genome Informatics Section, Computational and Statistical Genomics Branch, National Human Genome Research Institute, Bethesda, MD USA; 3 Dairy Forage Research Center, ARS USDA, Madison, WI USA; 4 Animal Genomics and Improvement Laboratory, ARS USDA, Beltsville, MD USA; 5 Phase Genomics, 4000 Mason Road, Suite 225, Seattle, WA 98195 USA; 6 Pacific Biosciences, Menlo Park, CA 94025 USA; 7 National Center for Biotechnology Information, National Library of Medicine, National Institutes of Health, Bethesda, MD 20894 USA; 8 European Molecular Biology Laboratory, European Bioinformatics Institute, Wellcome Genome Campus, Hinxton, Cambridge, CB10 1SD UK; 9 Center for Bioinformatics and Computational Biology, Lab 3104A, Biomolecular Science Building, University of Maryland, College Park, MD 20742 USA; 10 Bionano Genomics, San Diego, CA USA; 11 US Meat Animal Research Center, ARS USDA, Clay Center, NE USA

**Keywords:** Computational biology and bioinformatics, Animal breeding, Structural variation, Genomics

## Abstract

Inbred animals were historically chosen for genome analysis to circumvent assembly issues caused by haplotype variation but this resulted in a composite of the two genomes. Here we report a haplotype-aware scaffolding and polishing pipeline which was used to create haplotype-resolved, chromosome-level genome assemblies of Angus (taurine) and Brahman (indicine) cattle subspecies from contigs generated by the trio binning method. These assemblies reveal structural and copy number variants that differentiate the subspecies and that variant detection is sensitive to the specific reference genome chosen. Six genes with immune related functions have additional copies in the indicine compared with taurine lineage and an indicus-specific extra copy of fatty acid desaturase is under positive selection. The haplotyped genomes also enable transcripts to be phased to detect allele-specific expression. This work exemplifies the value of haplotype-resolved genomes to better explore evolutionary and functional variations.

## Abstract (web summary)

Taurine and indicine cattle have different desirable traits making them better adapted to different climates across the world. Here, Low et al. describe a pipeline to produce haplotype-resolved, chromosome-level genomes of Angus and Brahman cattle breeds from a crossbred individual and report on comparisons of the two genomes.

## Introduction

About 10,000 years ago, cattle were domesticated from the aurochs, which ranged across Eurasia and North Africa but are now extinct^1^. Modern day cattle belong to two subspecies, the humped zebu or indicine breeds (*Bos taurus indicus*) and the humpless taurine breeds (*Bos taurus taurus*), which arose from independent domestication events of genetically distinct aurochs populations^2^.

During the past century, taurine breeds have been intensively selected for production traits, particularly milk and meat yield, and generally have higher fertility than indicine breeds. European taurine breeds, such as Angus, have excellent carcass and meat quality, high fertility, and reach puberty early. These breeds have been imported by farmers around the world to improve or replace less-productive breeds. However, while European taurine animals are well adapted to temperate environments, they do not thrive in hot, humid tropical environments with high disease and parasite challenge.

Indicine breeds originated from the Indus valley and later spread to Africa and across southeast Asia^3^. Between 1854 and 1926, the four indicine breeds, Ongole, Krishna, Gir, and Gujarat, were imported into the United States and crossed with European taurine cattle to create the Brahman breed. Current US Brahman cattle retain ~10% of their genome of taurine origin^4^. Brahman have a short, thick, glossy coat that reflects sunlight and loose skin that increases the body surface area exposed for cooling. While Brahman are less productive and have lower fertility than taurine breeds, they have desirable traits, such as heat tolerance, lower susceptibility to parasites such as ticks, and are more disease and drought resistant^5^.

We previously demonstrated a trio binning approach to assemble haplotypes of diploid individuals at the contig level. The quality of the contigs exceeded those of the best livestock reference genomes^6^. Here we present chromosome-level taurine (Angus) and indicine (Brahman) cattle genomes from a single crossbred individual that were assembled with haplotype-aware methodology that is less laborious than sequencing haploid clones^7^. The contiguity and accuracy of the final haplotype-resolved cattle assemblies set a high standard for diploid genomes and enable precise identification of genetic variants, from single-nucleotide polymorphisms (SNPs) to large structural variants (SVs). A further benefit of haplotype-resolved genomes is that they can be used to better interpret allele-specific expression in diploid transcriptome profiles. We identify allele-specific and novel transcripts using PacBio Iso-Seq reads mapped onto the haplotype-resolved genomes. Considering the large differences in production and adaptation traits between taurine and indicine cattle, comparison of genomes between the breeds will contribute to unveiling the mechanisms behind phenotypic differences among cattle including environmental adaptation, which is of substantial scientific and economic interest.

## Results

### De novo assembly and annotation of Angus and Brahman genomes

The initial creation of haplotigs (haplotype-specific contigs) was presented in the description of the trio binning method implemented in TrioCanu^6^. Briefly, a male *Bos taurus* hybrid fetus, from an Angus sire and a Brahman dam, was sequenced to ~136× long-read coverage, and the reads were sorted into parental haplotype bins based on *k*-mers that are unique to either the paternal or maternal genome, which were identified by short-read sequencing of the parents prior to assembly with TrioCanu. The initial assemblies comprised 1747 Angus haplotigs and 1585 Brahman haplotigs (Table 1). The haplotig N50 was 29.4 and 23.4 Mb for the Angus and Brahman, respectively.

**Table 1:** Assembly statistics.

Breed	Assembly	Software	Assembly level	Number of sequences^a^	Number of gaps	N50 (Mb)	Assembly size (Gb)
Angus	PacBio	CANU	Haplotig	1747	0	29.4	2.6
Angus	PacBio+Hi-C	SALSA2	Scaffold	1515	235	104.6	2.6
Angus	PacBio+Optical map	Bionano Access	Scaffold	1595	181	35.2	2.6
Angus	UOA_Angus_1	PBJelly, Aarow, custom scripts	Chromosome	1435	277	102.8	2.6
Brahman	PacBio	CANU	Haplotig	1585	0	23.4	2.7
Brahman	PacBio+Hi-C	SALSA2	Scaffold	1370	216	72.6	2.7
Brahman	PacBio+Optical map	Bionano Access	Scaffold	1353	268	31.7	2.7
Brahman	UOA_Brahman_1	PBJelly, Aarow, custom scripts	Chromosome	1251	302	104.5	2.7

^a^There are 1405 and 1220 unplaced haplotigs in the final chromosome-level Angus and Brahman assemblies, respectively. These unplaced haplotigs comprise ~3.8% of total bases in the Angus assembly and ~2.1% of total bases in the Brahman assembly. Only the Brahman assembly has a complete mitochondrion sequence.

**Fig. 1:**
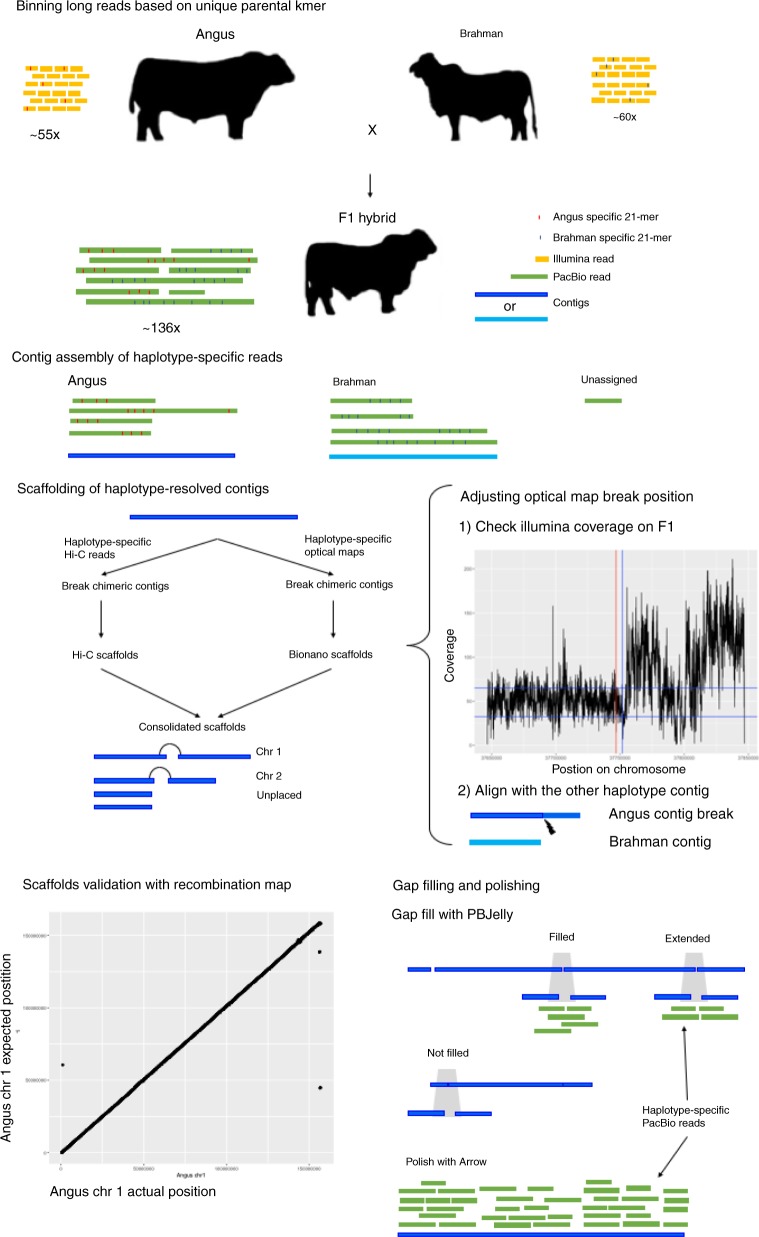
An overview of the assembly methods. Long PacBio reads were binned to the respective haplotypes using parental-specific *k*-mers and unassigned reads were discarded. TrioCanu was used to assemble sequences from each haplotype into haplotigs. Each set of haplotigs was scaffolded separately with both Hi-C and optical map data (illustrated only for the Angus). Optical map breakpoints were accepted but are imprecise. Therefore, breakpoint positions were improved by observing if there are local drops in short-read coverage and/or where there is a break sequence alignment with the alternative haplotig. Hi-C and optical map-based scaffolds were checked for consistency and combined as a single set of scaffolds. Cattle recombination maps were used to validate the assembly. Each point on the scatter plot is the actual recombination marker coordinate on the latest reference genome and the expected position based on previous reference genome, UMD3.1. Finally, haplotype-specific long reads were used to fill gaps and polish the sequence.

For the present study, additional data were generated for the same hybrid fetus, including ~12× Hi-C reads, ~167× Bionano optical map, and ~84× Illumina paired-end reads (Fig. 1), to provide haplotype-resolved scaffolding and identify assembly errors. Following haplotig assembly, two sets of scaffolds, one based on Hi-C and the other on optical map data, were generated for each haplotype. Three different scaffolding programs (3D-DNA, Proximo, and SALSA2) were evaluated using the Hi-C data (Supplementary Note 1). SALSA2 was found to be the best scaffolder and produced the closest agreement with the latest cattle reference ARS-UCD1.2. The scaffold N50 produced by SALSA2 was larger than that generated by optical map scaffolding, but the latter detected chimeric haplotig more accurately (i.e., a haplotig incorrectly assembled), which resulted in 29 and 36 breaks in the Angus and Brahman haplotigs, respectively (Supplementary Note 2). These chimeric breaks corrected four inter-chromosomal fusions in the initial haplotigs, involving two Brahman chromosomes (13 and 15) and six Angus chromosomes (8, 9, 12, 20, 23, 28).

After validation against a recombination map, gap filling, and error correction, the final assemblies, UOA_Angus_1 and UOA_Brahman_1, had chromosome sizes similar to the current cattle reference, ARS-UCD1.2 (Supplementary Fig. 1). Unlike some of the recent PacBio-based assemblies^8, 9^, which required an additional polishing step with Illumina short reads to correct the high indel error rates, the haplotype-resolved assemblies only required correction of a very small number of coding sequences, showing that polishing with short reads was unnecessary (Supplementary Note 3).

The Brahman genome was annotated by Ensembl and NCBI, whereas the Angus genome was annotated only by Ensembl (Supplementary Notes 3 and 4). A comparison of annotation features between the Angus, Brahman, and Hereford reference genomes is given in Supplementary Table 1. As the Ensembl pipeline was used to annotate all three cattle genomes, interpretation of results reported here used Ensembl release 96.

### Assembly benchmarking and sequence contiguity assessments

The per-base substitution quality values (QVs) for the UOA_Angus_1 and UOA_Brahman_1 reference assemblies were 44.63 and 46.38, respectively (Supplementary Table 2, Supplementary Note 5). The QV represents the phred-scaled probability of an incorrect base substitution in the assembly, hence these QVs indicate that the assemblies are >99.99% accurate at single base level. This is similar to the latest water buffalo assembly UOA_WB_1 (QV 41.96) and surpasses the recent goat ARS1 assembly (QV 34.5) by an order of magnitude. The Angus and Brahman assemblies had ~93% BUSCO completeness score, which demonstrates a high-quality (HQ) assembly of genes (Supplementary Table 3).

**Fig. 2:**
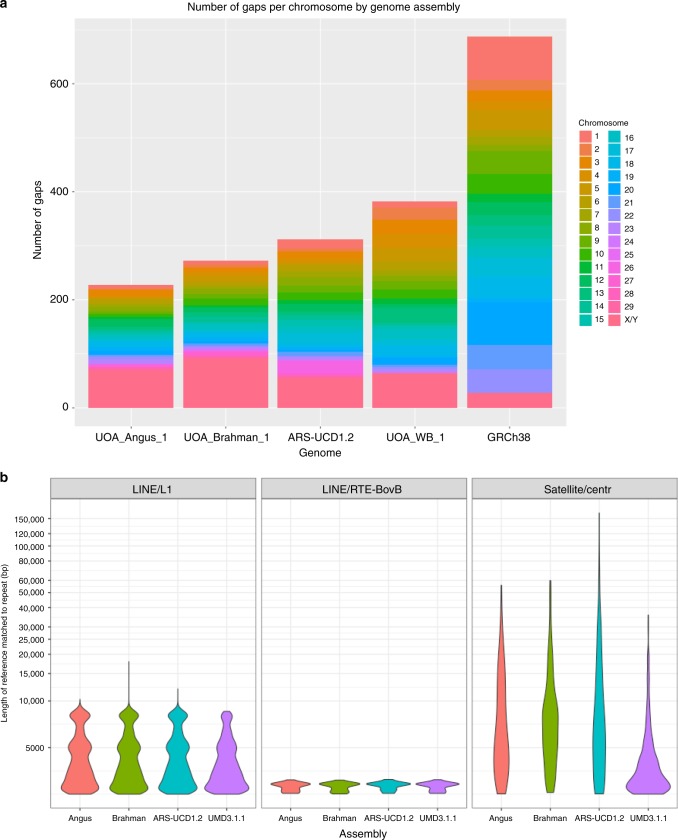
Sequence contiguity and resolution of repeats. **a** Barplot of the number of gaps by chromosomes between various mammalian assemblies. **b** Violin plot of repeat families filtered for those >2.5 kb for LINE/L1, LINE/RTE-BovB, and satellite/centromeric repeats.

The Angus and Brahman assemblies have few gaps compared to most existing mammalian reference assemblies and are comparable to the human GRCh38, the latest Hereford cattle ARS-UCD1.2, and the water buffalo UOA_WB_1 reference genomes (Fig. 2a). For example, the Angus chromosome 24 was assembled without gaps. In terms of contiguity, these cattle reference genomes are comparable to the recent water buffalo UOA_WB_1 assembly^9^, which is the most contiguous ruminant genome published to date with <1000 contigs (Supplementary Fig. 2), although it is not fully haplotype resolved. While the cattle autosomes showed excellent contiguity, the Brahman X and Angus Y chromosomes were interrupted by 91 and 69 gaps, respectively.

### Resolution of longer repeats

The use of long PacBio reads substantially improved repeat resolution compared with the previous cattle assembly UMD3.1.1, which was assembled from Sanger sequences^10^ (Fig. 2b). Approximately 49% of both Angus and Brahman assemblies consist of repeat elements, which is consistent with other published mammalian assemblies, including human GRCh38, Hereford cattle ARS-UCD1.2, water buffalo UOA_WB_1, and goat ARS1. The two largest repeat families identified were Long Interspersed Nuclear Element (LINE) L1 and LINE/RTE-BovB, which covered ~25% of the chromosomes in both cattle sub-species (Supplementary Fig. 3). Satellite or centromeric repeats (>10 kb) accounted for 21% and 14% of repeats in unplaced scaffolds of Angus and Brahman, respectively. The 7% higher satellite and centromeric repeats in Angus unplaced scaffolds may be due to the presence of the Y chromosome in the Angus haplotype. The combination of the three most frequent repeat families, LINE L1, LINE/RTE-BovB, and satellite/centromeric repeats, covered ~40% of all unplaced bases, and repeat sequences were most frequently responsible for breaking sequence contiguity. The three cattle assemblies constructed using PacBio long reads that resolved repeats >2.5 kb, UOA_Angus_1, UOA_Brahman_1, and ARS-UCD1.2, provide significant improvements in repeat resolution over the previous Sanger-based cattle assembly (UMD3.1.1) (Fig. 2b). In both the Brahman and Angus assemblies, 20 out of 29 of the autosomes contained centromeric repeats within 100 kb of chromosome ends. Vertebrate telomeric repeats (TTAGGG)n were found within 1 Mb of the ends of six Angus and five Brahman chromosomes. This demonstrates that some scaffolds approach chromosome-level assembly.

### Discovery of indicus-specific fatty acid desaturase 2

**Fig. 3:**
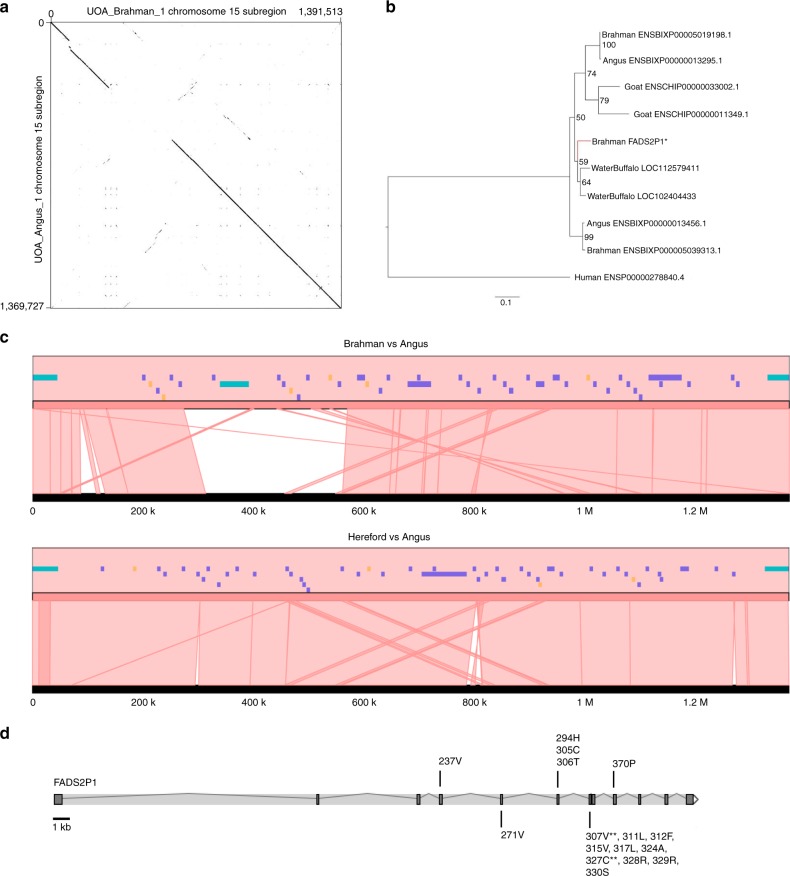
Divergence of the FADS2P1 locus between indicine and taurine cattle. **a** Dot plot of Brahman chromosome 15 between positions 3,748,952 to 5,140,465 against the homologous Angus chromosome between positions 78,799,177 to 80,168,904. The Brahman sequence was reverse complemented in the plot. **b** Maximum likelihood tree with 1000 bootstraps of FADS2P1 homologous protein sequences. The extra Brahman FADS2P1 copy is highlighted with asterisk (*) and its branch colored red. **c** Microsynteny plot showing a lack of sequence conservation between indicine and taurine breeds around the indicine-specific *FADS2P1* gene. All *FADS2P1* genes are colored turquoise, other genes purple, and pseudogenes orange. The upper plot compares Brahman to Angus and the lower plot compares Hereford to Angus. The track in black in both panels is the Angus reference. The Brahman *FADS2P1* gene Ensembl IDs are ENSBIXG00005007613, ENSBIXG00005021668, and ENSBIXG00005022680, whereas the Angus IDs are ENSBIXG00000018262 and ENSBIXG00000018381. The indicus-specific copy of *FADS2P1* is ENSBIXG00005007613. **d** Mapping of 16 positively selected sites onto the exons of Brahman *FADS2P1*. The residues with double asterisks (**) indicate they have ${\rm{Prob}}\left( {\omega \,> \, 1} \right) \,> \, 0.99$ (i.e., highly significant positively selected sites).

One of the most diverged genomic regions between Brahman and Angus was observed on chromosome 15 (Fig. 3a). A region of ~1.4 Mb has three copies of fatty acid desaturase 2-like genes (*FADS2P1*) in Brahman, whereas the homologous region in the Angus only has two *FADS2P1* genes (Fig. 3b, c). In both Brahman and Angus, the *FADS2P1* genes are encoded by 10–12 exons, and the entire region was assembled completely without gaps for both genomes. The region also contains six genes annotated as olfactory receptor-like, with unknown functions, which had differences in their predicted gene models between Brahman and Hereford assemblies. Within the ~1.4 Mb region, there is a high level of sequence divergence for ~200 kb, which is where an extra copy of *FADS2P1* lies in Brahman. Searches for *FADS2P1* in other ruminant species with HQ genome assemblies revealed that only Brahman has three copies of the gene. The additional *FADS2P1* gene is ~53 kb long and is flanked by two other conserved *FADS2P1* genes. Searching whole-genome sequencing (WGS) short-read sequences from 38 animals used in this study showed that only Brahman animals had the extra copy, which was not present in any of the taurine individuals. (Supplementary Fig. 4). Considering that the Brahman genome is derived from four indicine breeds, the extra *FADS2P1* is likely a *Box taurus indicus*-specific gene. We used a maximum likelihood-based estimate of ratio of non-synonymous (amino acid changes) to synonymous (silent changes) substitutions as implemented in CODEML to search for positively selected amino acid residues in FADS2P1 and identified 16 significant positively selected sites, 10 of which are located in a small exon 7 of only 60 bp (Fig. 3d, Supplementary Table 4).

### SNP and INDEL differences between Brahman and Angus

Mapping short reads from Brahman and Angus to both reference genomes, UOA_Brahman_1 and UOA_Angus_1, revealed that the use of breed-specific reference genomes gave a lower count of all classes of genetic variants. Using WGS short reads from 5 Brahman and 6 Angus individuals, we identified ~24 million Brahman SNPs and ~11 million Angus SNPs, which were annotated using their own reference genome (Table 2, Supplementary Table 6). There were about twice as many INDELs in the Brahman (2,804,421 bp) than the Angus (1,381,548 bp) samples. Lower counts of SNPs, INDELs, and the four classes of SVs (i.e., BND, DEL, DUP, INV) were identified when the appropriate reference genome was used. For example, ~4% fewer SNPs were observed when Brahman individuals were mapped onto the Brahman instead of the Angus reference genome. Additional information on the use of SNPs for the analysis of selective sweeps in cattle is given in Supplementary Note 6.

**Table 2:** Polymorphism statistics.

Breed^a^	Reference	SNP	INDEL	BND	DEL	DUP	INV
Angus	Angus	10,615,122	1,381,548	38	84	22	3
Brahman	Angus	24,930,357	2,928,526	311	641	86	22
Angus	Brahman	16,504,067	2,090,735	159	182	40	11
Brahman	Brahman	23,876,357	2,804,421	279	481	97	18

*BND* complex structural variant, *DEL* deletion, *DUP* duplication, *INV* inversion.

^a^Breed here refers to the ~10× WGS short reads used to align to the reference. BND, DEL, DUP, and INV are structural variant types called in Lumpy.

### SV differences between Brahman and Angus

We assessed the structural continuity of our Brahman and Angus genome assemblies against the current cattle reference genome assembly, ARS-UCD1.2, and against WGS datasets from 38 animals representing seven breeds, to ascertain the benefit of using haplotype-resolved assemblies for variant calling. To assess SV differences between Brahman and Angus and the cattle reference genomes, the haplotype-resolved assemblies were aligned to the ARS-UCD1.2 reference (Hereford). This detected insertions, deletions, tandem expansions, tandem contractions, repeat expansions, and repeat contractions^11^ (Supplementary Fig. 5). Both tandem expansion/contraction and repeat expansion/contraction are repeat-type SVs. Detection of SVs was limited to sizes of 50–10,000 bp, and the total bp affected by SVs in Angus and Brahman were 10.9 and 21.8 Mb. This translates to approximately 0.4% and 0.8% of the Angus and Brahman genomes, respectively. Among the six classes of SVs examined, insertion/deletion types were the most prevalent in both Brahman and Angus genomes compared to ARS-UCD1.2.

**Fig. 4:**
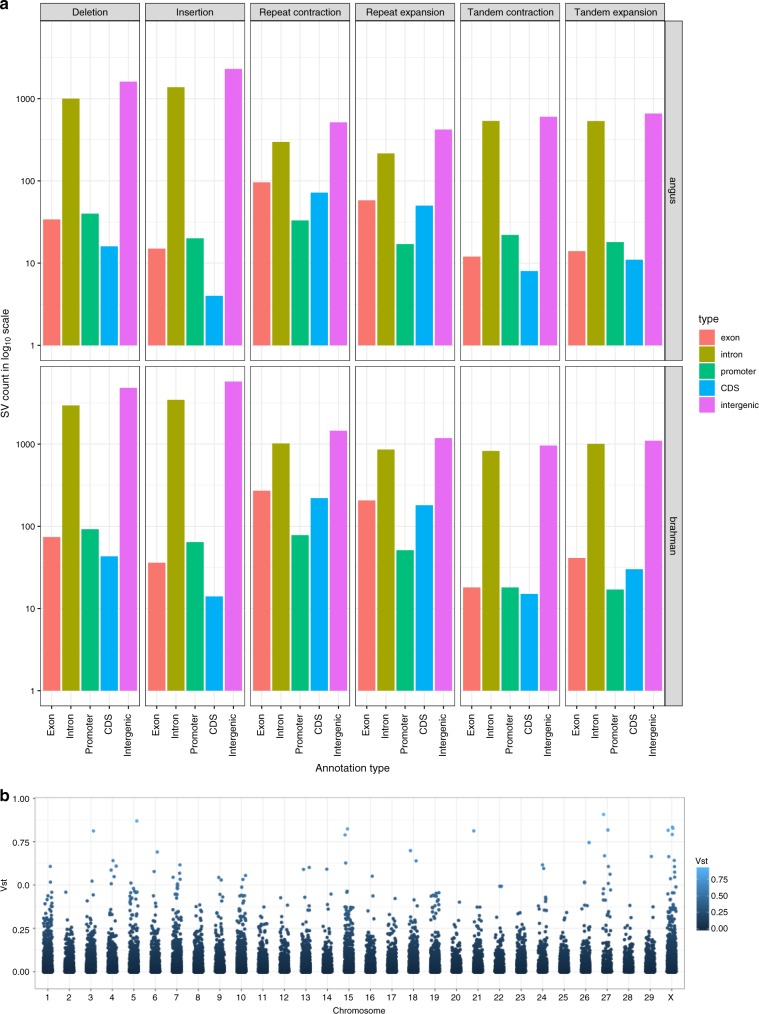
Comparison of structural variants between Brahman and Angus. **a** Count in log_10_ scale of 6 classes of SVs when overlapped with various annotation types. **b** Population differentiation for copy number variation as estimated by *V*_st_ along each chromosome for the taurine and indicine comparison using UOA_Brahman_1 as the reference.

We extracted Brahman- and Angus-specific SVs to study their distribution in genic and intergenic regions (Fig. 4a). Brahman-specific insertions/deletions overlapped ~4% of all genes, whereas Angus-specific insertions/deletions overlapped only ~1–2% of genes. Each repeat-type SV overlapped ~1% of genes in Brahman and <1% in Angus. The majority of SVs were found in intergenic regions, and when they overlapped with genes, they were generally localized within introns. Over-representation of Gene Ontology (GO) terms was detected for Angus-specific insertions and tandem contractions and Brahman-specific insertion/deletion SVs at false discovery rate (FDR)-adjusted, Fisher’s exact test, *P* value < 0.05 (Supplementary Table 7). No over-representation of GO terms was detected for any of the other breed-specific SV types. Interestingly, Brahman-specific insertion SVs have between 3- and 5.7-fold enrichment in phospholipid translocation (GO:0045332), lipid translocation (GO:0034204), lipid transport (GO:0006869), and lipid localization (GO:0010876) GO classes, which suggests that lipid distribution was most impacted by SVs.

**Fig. 5:**
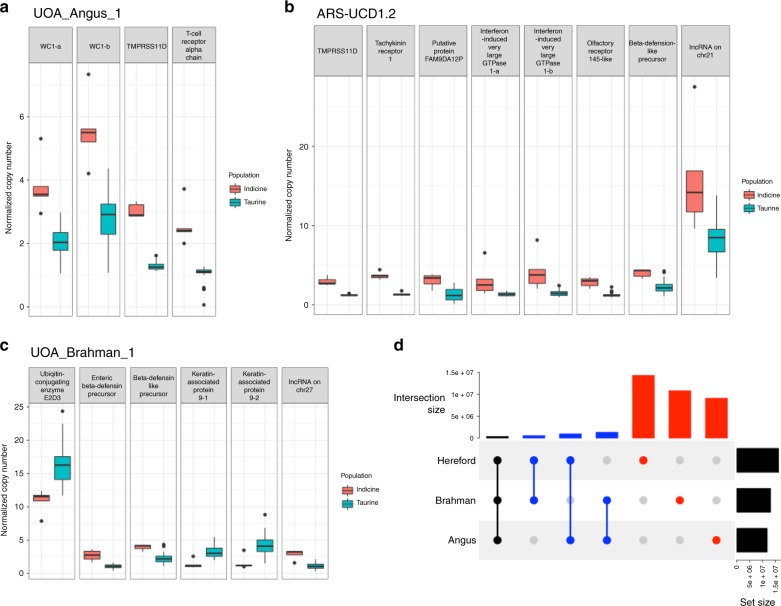
Boxplot of normalized copy number of autosomal genes with *V*_st_ > 0.3. Only those CNV genes with average copy number difference of at least 1.5 copies between the taurine and indicine groups are shown. Dot plots of individual values are overlaid on top of boxplots to show minima and maxima as circles. The bounds of box show the 25th and 75th percentile, with the median drawn as a thick line between these two quartiles. The reference genomes were **a** UOA_Angus_1, **b** ARS-UCD1.2, and **c** UOA_Brahman_1. **d** Liftover of CNV regions from Brahman and Angus to Hereford ARS-UCD1.2 common coordinate for an assessment of intersection between them at base-pair resolution.

Using WGS reads from different datasets, we identified subspecies-specific copy number variations (CNVs) that were masked by the absence or poorer resolution of sequence in the ARS-UCD1.2 reference. The input dataset for these analyses came from ~10× WGS short reads of 38 animals representing 7 cattle breeds. Each set of reads was aligned to all three reference genome assemblies (Hereford, Brahman, and Angus) and processed with SV callers designed to detect read depth differences and paired-end/split-read (PE) discordancy, respectively. The read-depth variation approach included the use of the *V*_st_ statistic^12, 13^ to identify genes with CNV between taurine or indicine lineages using the Brahman, Angus, or ARS-UCD1.2 assemblies (Fig. 4b, Supplementary Fig. 6). The values of *V*_st_ varied greatly depending on which reference genome was used for alignment, with taurine-based reference assemblies showing higher variance in copy number between the taurine and indicine lineage datasets (Supplementary Fig. 6) than the Brahman reference (Fig. 4b). Only autosomes were considered. Six CNV genes were found in Brahman, whereas four and eight CNV genes were found in Angus and Hereford, respectively (Fig. 5a–c). Prediction of CNV genes was sensitive to the assembly chosen, e.g., only *TMPRSS11D* and beta-defensin-like precursor were found to be copy number variable in more than one assembly. Among the 18 CNV genes differentiating indicine from taurine genomes, six unique gene families were identified, which were beta defensin, workshop cluster, trypsin-like serine protease, T cell receptor alpha chain, tachykinin receptor, and interferon-induced very large GTPase, all of which have immune-related functions. All of the CNV genes from these six families showed higher copy number in the indicine cattle lineage regardless of the assembly used. Intersection of liftover CNV regions (CNVRs) called using the Brahman assembly with repetitive elements on ARS-UCD1.2 showed a higher prevalence of CNVs that may have resulted from repeat expansion/contraction in the Brahman reference (1813) than in ARS-UCD1.2 (1238) or the Angus (1164) assemblies. FRC_align statistics showed a higher count of COMPR_PE and STECH_PE events in the Angus assembly (319 and 101, respectively) than the Brahman assembly (263 and 87, respectively), supporting the hypothesis that expansion and contraction of genomic sequence in the Angus assembly is the likely reason for these discrepancies. An olfactory receptor, two long non-coding RNAs and one putative protein, FAM90A12P, also had higher copy numbers among indicine animals. In contrast, ubiquitin-conjugating enzyme E2D3 and two keratin-associated protein 9 genes (*KRTAP9-1*, *KRTAP9-2*) had higher copy numbers in the taurine lineage.

We quantified the effects of using different reference assemblies for PE SV discovery. All SV calls of this type were converted into Hereford coordinates to facilitate comparisons. We removed 17, 9, and 18 PE SVs of all types from the Brahman, Angus, and Hereford assemblies that were likely false positives, as they were >1 Mb and did not correspond to aberrant read depth signal to support their SV calls. On average, 0.5% of each cattle genome was covered by CNVRs (Fig. 5d). The majority of CNVRs (at least 76% from each assembly) were found to be unique to one assembly. Among the Brahman CNVRs, only 10% intersected with Angus CNVRs, which suggests mis-assembly in the Hereford reference potentially due to compression of repetitive elements that are more difficult to resolve without phasing haplotypes using the trio binning method.

### Allele-specific transcripts in haplotype-resolved genomes

**Fig. 6:**
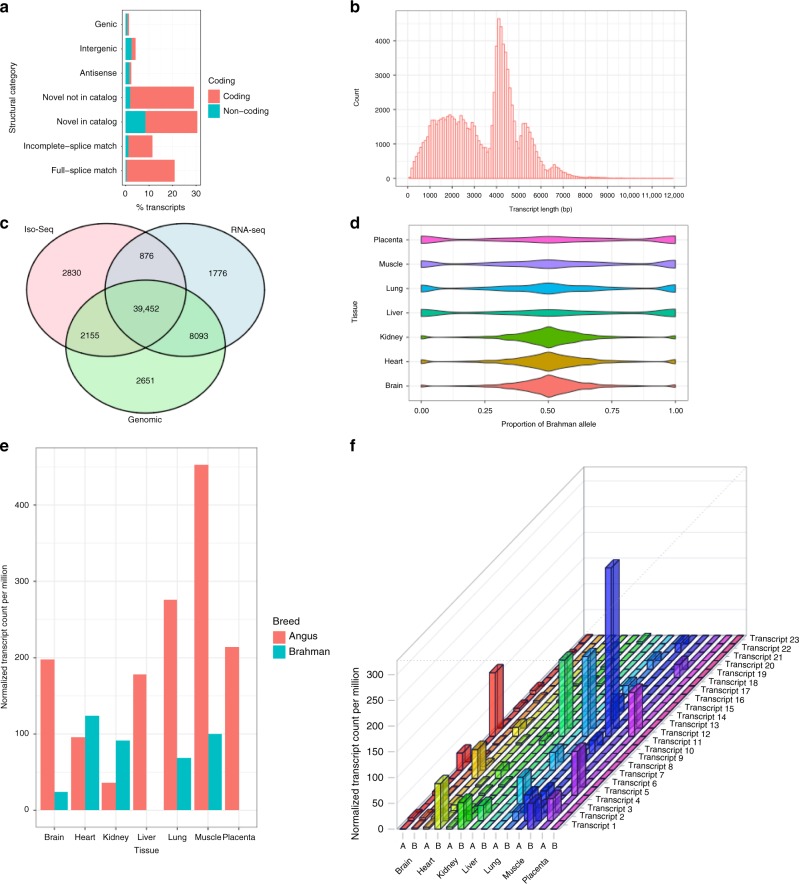
Phasing of Iso-Seq full-length transcripts in seven tissues reveals transcriptional complexity and allelic imbalance. **a** Characterization of transcript annotation of the hybrid animal using SQANTI2 against the Brahman annotation. Full-splice match: perfect match with a reference; incomplete-splice match: missing one or more 5’ exons against a reference; novel in catalog: novel combinations of known junctions; novel not in catalog: at least one novel splice site. **b** Histogram of transcript length distribution. **c** The overlap of SNPs between WGS short reads from genomic DNA, Iso-Seq, and RNA-Seq when Brahman was used as the reference genome. **d** Violin plot of the proportion of Brahman alleles, which was calculated as the normalized count of Brahman alleles divided by the sum of the normalized count of both Brahman and Angus alleles. Transcripts showing allelic imbalance and with higher expression in Brahman have values closer to 1, whereas those with higher expression in Angus have values closer to 0. **e** Tissue-specific allelic expression at the gene level for *ARIH2*, which is the most highly expressed Angus gene in the brain. **f** Tissue-specific allelic expression at the transcript level for *ARIH2* in the brain, heart, kidney, liver, lung, muscle and placenta. A denotes Angus and B denotes Brahman.

Among the PacBio error corrected Iso-Seq (circular consensus sequence (CCS)) reads pooled from seven tissues of the F1 hybrid fetus, 3,275,676 reads (55%) were classified as full-length non-concatamer (FLNC) reads. After processing with the isoseq3 software, 193,974 full-length, HQ consensus transcripts were generated. We mapped the HQ transcripts to the Brahman reference and obtained 99,329 uniquely mapped transcripts covering 20,940 non-overlapping loci representing 19,403 genes. Of these 99,329 Iso-Seq transcripts, 20,708 (20.8%) had a perfect exon-by-exon match to the reference annotation while 11,359 (11.4%) matched a reference transcript but was missing one or more of the 5’ exons (indicator of 5’ degradation or alternative start site). The majority of the remaining transcripts (59,158, 60%) were novel isoforms of known genes, with the remainder 6.8% of transcripts categorized as intergenic, genomic, or anti-sense that are likely cDNA artifacts. At the gene level, 13,754 of the 19,403 (71%) genes are annotated reference genes. Using the SQANTI2 transcript characterization tool, 83% of the Iso-Seq transcripts fell into coding regions of the Brahman annotation (Fig. 6a). The transcript length distribution ranged from 85 to 11,872 bp, with a median of 3853 bp and a mode of ~4 kb (Fig. 6b).

We validated the IsoPhase SNPs using (1) SNPs called from RNA-Seq data of the brain, liver, lung, muscle, and placenta of the F1 hybrid and (2) Angus SNPs derived from mapping Illumina WGS short reads of the F1 hybrid to the Brahman reference. As the RNA-Seq had greater coverage than Iso-Seq and the SNPs called from genomic DNA included non-transcribed regions, only SNPs that were in positions covered by at least 40 full-length Iso-Seq reads were retained. The concordance of filtered SNPs called from WGS, RNA-Seq, and Iso-Seq is very high (87%) (Fig. 6c). Of the 45,313 SNPs called by IsoPhase, 39,452 (87%) were validated by SNPs from RNA-Seq and WGS, whereas 876 (1.9%) were only validated by RNA-Seq and 2155 (4.7%) were only validated by WGS (Supplementary Note 7). SNP calls that showed inconsistencies could often be explained by lower Iso-Seq coverage, SNPs in homopolymer regions, or alignment artifacts.

Our haplotype-resolved genomes allowed us to explore genes with allelic imbalance in expression. To assess allelic imbalance, the proportion of an allele from each breed was calculated as the normalized count of the Brahman allele divided by the sum of normalized counts of both Brahman and Angus alleles. All tissues showed evidence of imbalance in allelic expression (Shapiro test, *P* value < 0.01), which was most pronounced for liver, lung, muscle and placenta, whereas brain, heart and kidney were less affected (Fig. 6d). However, as the mammalian brain consists of a wide range of cell types and hence transcriptional complexity, brain tissue was chosen to demonstrate the phasing of transcripts to explore allele-specific expression. The most highly expressed Angus gene with allelic imbalance (ratio of 8 Angus:1 Brahman) in the brain was *ARIH2* (also known as *TRIAD1*), which is known to play a role in protein degradation via Cullin-RING E3 ubiquitin ligases^14^ (Fig. 6e, f). *ARIH2* expression in the liver, lung, muscle, and placenta was also higher from the Angus allele than the Brahman or maternal allele. The HQ transcripts included 23 different transcript isoforms of *ARIH2*; however, 66% of transcripts for this gene across the 7 tissues were represented by only 3 isoforms. The annotated exons of this gene were in good agreement with the RNA-Seq data (Supplementary Fig. 7).

The most highly expressed Brahman gene with allelic imbalance (ratio of 1 Angus:6 Brahman) in the brain was Calmodulin (*CaM*), a heat-stable Ca^2+^-binding protein that mediates the control of numerous physiological processes, including metabolic homeostasis, phospholipid turnover, ion transport, osmotic control, and apoptosis^15^ (Supplementary Fig. 8). Surprisingly, we also found allelic imbalance (ratio of 1 Angus:16.5 Brahman) in pregnancy-associated glycoprotein 1 (*PAG1*) with a higher expression of the Brahman allele in the brain and placenta but undetectable in other tissues. This gene was previously thought to be placenta specific and is used as a biomarker for embryo survival^16^.

## Discussion

Traditional genome assembly approaches collapse haplotypes and therefore do not allow accurate assembly or the study of divergent, heterozygous regions. Here we demonstrate an assembly approach that yielded highly contiguous, haplotype-resolved Brahman and Angus cattle genomes from an F_1_ hybrid of the two subspecies. Our analyses demonstrated that previous studies^4, 17^, which mapped indicine sequences onto the taurine reference UMD3.1.1, will have identified loci where the subspecies are fixed for different alleles. Calling SNPs in transcripts from a diploid hybrid with both haplo-genomes decoded provides accurately phased transcripts for studies on the role of allele-specific expression in, e.g., hybrid vigor or heterosis. The phasing of Iso-Seq transcripts in reciprocal crosses will facilitate the exploration of breed-specific effects on parental imprinting, which has been shown in maize^18^.

We found that the choice of reference assembly had a large impact on SV calling. The observed SV difference between Brahman and Angus is in part due to using Hereford as the reference, which is more closely related to Angus. Ambiguous read alignments, which result from the assignment of reads to incorrect positions on the genome, are a major factor in SV call accuracy^19^. This is a major concern in the detection of variant sites from alignment of reads to fasta-based reference assemblies. This has prompted the creation of graph-based file formats to improve alignment accuracy^20, 21^. After converting SVs from each assembly onto the Hereford assembly coordinates and calculating the intersection, we identified 1.3 Mbp of SVs (33% in genic regions) present in the Angus and Brahman assemblies that were not present in the Hereford assembly. This suggests that either the Hereford assembly was not as representative of the true structural variation in these regions or that there were assembly errors in the Angus and Brahman assembly that generated false positive SVs. The latter is less likely given the high accuracy of the Angus and Brahman genomes. *V*_st_ estimates for copy number windows incorrectly determined heightened variance between taurine and indicine animals on chromosome 15, suggesting that comparative alignment approaches are prone to a high FDR when used to detect true structural differences between species or subspecies of cattle. If only one reference genome is available for a genus or species, this could present a substantial issue in the interpretation of comparative SV analysis. Conversely, we identified 0.9 Mbp SVs shared between only the Hereford and Angus assembly, which may represent true genomic structural differences between taurine and indicine cattle.

HQ haplotype-specific assemblies facilitate genome-wide comparisons to identify novel variation. The discovery of an indicus-specific, additional copy of *FADS2P1*, which has been under positive selection, is an example that highlights the benefits of HQ haplotype-specific assemblies. The *FADS2P1* gene region in both Brahman and Angus span ~1.4 Mb of sequence, while the two *FADS2P1* genes in the water buffalo span ~1 Mb. The orthologous region in goat is ~1 Mb but contains gaps. Taking phylogenetic and information on conservation of synteny together, the most parsimonious explanation is that the extra *FADS2P1* was duplicated in the indicine lineage after divergence from taurine cattle. Rapid evolution at the *FADS2P1* locus resulted in neofunctionalization of the additional gene in indicine animals, with profound changes seen in the small exon 7.

*FADS2* is a pleiotropic gene with known functions in the biosynthesis of unsaturated fatty acids, lipid homeostasis, inflammatory response, and promotion of myocyte growth and cell signaling^22–24^. A non-synonymous SNP in exon 7 of Japanese Black cattle is significantly associated with linoleic acid^25^ composition. While we do not know the functional significance of positively selected residues in the additional *FADS2P1* copy in Brahman, the SNP reported in the Japanese Black shows the importance of exon 7 in FADS2 function. Studies in rats have shown that linoleic acid is an important component of skin ceramides and its deficiency increases water permeability of the skin^26^. Comparisons between indicine and taurine animals have shown differences in fatty acids^27^ and types of phosphatidylcholines^28^. We hypothesize *Bos indicus* has three copies of *FADS2P1* genes to regulate the composition of fatty acids that constitute the cell membranes and could alter water permeability and heat loss from skin.

Brahman cattle may be better adapted to harsher environments because they have slower protein turnover^29^. Relative to Angus, Brahman have much lower expression of *ARIH2* in key metabolic organs, such as the skeletal muscle, and no detectable expression in the liver. ARIH2 promotes ubiquitylation of DCNL1, which is a co-E3 ligase that performs cullin neddylation, a process that regulates one-fifth of ubiquitin-dependent protein turnover^14^. CNV analysis revealed a decreased number of ubiquitin-conjugating enzyme E2D3 genes in the indicine lineage, which suggests lower protein turnover in indicine animals. While it is still speculative, our findings are consistent with lower protein turnover and the ability of Brahman cattle to withstand stressful conditions.

The analyses of CNV by alignment of short-read sequences from 38 individuals from 7 breeds to the Brahman and Angus genomes revealed that 6 genes with immune-related functions and putative roles in response to disease challenge and external parasites have additional copies in the indicine lineage. Conversely, *KRTAP9-*2, a gene with significantly altered gene expression following tick infestation^30^, is expanded in the taurine lineage, which has also been reported in previous CNV studies^13, 31^. Further studies are needed to elucidate how changes in copy number of *KRTAP9-*2 affect its expression and its role in tick resistance.

In conclusion, the approach used here is able to create haplotype-resolved genome assemblies that are of higher quality than traditional haplotype-collapsed assemblies. Availability of these HQ assemblies has enabled us to better resolve SVs and identify regions under selection that may be involved in adaptation to the environment. Looking forward, it is clear that HQ haplotype-resolved assemblies together with long-read transcript information will underpin studies on genome function, regulation, and the control of phenotypes.

## Methods

### *Bos taurus* hybrid

A *Bos taurus indicus* female (Brahman) was inseminated with semen from a *Bos taurus taurus* (Angus) bull. The *indicus* maternal genetic background of the Brahman dam was confirmed by mitochondrial DNA haplotype analysis^32^. At day 153 post-insemination, dam and conceptus were ethically sacrificed and fetal brain, heart muscle, kidney, liver, lung, skeletal muscle, and placenta (cotyledon) tissue were snap frozen in liquid nitrogen and stored at −80 °C until further use. All animal work was approved by the Animal Ethics Committee of the University of Adelaide (No. S-094-2005).

### Genome sequencing and assembly of contigs

DNA was extracted from fetal lung using a salting out method^6^. Briefly, 100 mg of tissue was ground into powder under liquid nitrogen and then transferred to a tube containing nuclei lysis solution (2 ml buffer of 10 mM Tris-HCl pH 8, 0.4 M NaCl, 2 mM EDTA, 0.2 ml 10% sodium dodecyl sulfate, 0.06 ml 10 mg/ml RNase A). After mixing at 37 °C for 1 h, 0.025 ml of Proteinase K (20 mg/ml) was added to the solution and shaken overnight, and DNA was precipitated by salting out. The dam uterus and bull semen DNA were extracted using standard phenol–chloroform procedures. Twelve SMRT sequencing libraries were made from the fetal DNA using the protocol recommended by the Pacific Biosciences (Procedure P/N 100-286-000-07), with a 15-kb size selection cut-off on a Blue Pippin instrument (Sage Science, Beverley, MA). Nine libraries were sequenced using P6/C4 chemistry on an RSII machine, whereas the remaining three libraries were sequenced on a Sequel machine. Approximately 161 Gb of RSII data and 205 Gb of Sequel data were produced, which gave a total sequence yield of 366 Gb with the mean read length of ~10.4 kb. Assuming a genome size of 2.7 Gb, the raw PacBio data represents ~136× coverage.

Illumina sequencing libraries for both parents (i.e., sire and dam) and F1 fetus were prepared using TruSeq PCR-free preparation kits (Illumina, San Diego, CA). A total of ~55×, ~60×, and ~84× coverage of 150 bp paired-end reads were generated for the sire, dam, and F1 fetus, respectively. In order to assemble phased haplotigs for the F1 Brahman–Angus hybrid, we used the trio binning method introduced by Koren et al.^6^. Briefly, 21-mers were identified in both sire and dam Illumina reads and 21-mers unique to one or other parent were used to assign the F1 PacBio long reads to the parent of origin. Approximately 1% of the PacBio reads were excluded from the assembly as they lacked parent-of-origin-specific 21-mers, due to their shorter lengths (Supplementary Fig. 9). Long reads that were binned into paternal and maternal groups were assembled separately with TrioCanu v1.6.

### Hi-C library preparation and sequencing

A Sau3AI Hi-C library was prepared (Phase Genomics, Seattle, WA) as follows: approximately 200 mg of fetal lung tissue was finely chopped and then cross-linked in Proximo crosslinking solution. The 5’ overhangs after Sau3AI digestion were filled with biotinylated nucleotides, and free blunt ends were ligated. After ligation, crosslinks were reversed and the free DNA was column purified and sonicated to approximately 600 bp peak fragment size (Bioruptor, Diagenode). Hi-C junctions were bound to streptavidin beads and washed to remove unbound DNA. Washed beads were used to prepare sequencing libraries using the HyperPrep Kit (Kapa) following the manufacturer’s protocols. In total, 203 million 2 × 81 bp read pairs were sequenced on NextSeq Illumina platform.

### Scaffolding of contigs with Hi-C

All Hi-C reads were mapped to each breed-specific set of haplotigs using BWA v0.7.15^33^. A haplotype score for a pair was defined as the sum of the percent identity multiplied by match length for each read end (unmapped read ends were assigned a score of 0). Each read pair had two scores, one per haplotype. Pairs with a higher score for one haplotype were considered breed specific and assigned to their respective haplotype. Pairs with a tied score were considered homozygous and assigned to both haplotypes for scaffolding.

Three different Hi-C based scaffolding programs, 3D-DNA^34^, Proximo (Phase Genomics), and SALSA2^35^, were evaluated for scaffolding contigs. Further detail on the comparison between the scaffolders is given in Supplementary Note 1. Reads were mapped with the Arima mapping pipeline (https://github.com/ArimaGenomics/mapping_pipelinecommit72c81901c671203a86ca4675457004a71d0cd249) and converted to bed format prior to SALSA2 scaffolding (https://github.com/machinegun/SALSAgitcommit863203dd094aaf9b342c35feedde7dabeec37b44), which was run with parameters -c 10000 -e GATC -m yes, for both breed-specific haplotigs.

### Bionano DNA isolation and assembly

DNA was extracted from 10 mg kidney tissue from the F1 hybrid using the Bionano Animal Tissue DNA Isolation Kit (P/N 80002) with slight modifications as follows: the frozen tissue was crushed in liquid nitrogen, placed in 2% formaldehyde in Bionano animal tissue homogenization buffer (Document number 30077, Bionano-Prep-Animal-Tissue-DNA-Isolation-Soft-Tissue-Protocol.pdf), and blended with a rotor-stator. The homogenate was passed through a 100-μm nylon filter, fixed on ice for 30 min in 2 ml 100% ethanol, and centrifuged for 5 min at 2000 × *g*. The resulting pellet was re-suspended in homogenization buffer and added to pre-warmed agarose to make 0.8% agarose plugs. High molecular weight DNA was extracted from the agarose plugs, labeled, stained, and imaged on a Bionano Saphyr system^36^. Further detail on de novo optical map assembly is given in Supplementary Note 2.

### RNA-Seq and Iso-Seq

RNA was extracted from tissue and ground to a fine powder under liquid nitrogen using the Qiagen RNeasy Plus Universal Kit as per the manufacturer’s instructions. RNA quality was assessed using an Agilent TapeStation system and confirmed as RNA integrity number >8 for all samples. Sequencing libraries were prepared with the KAPA Stranded RNA-Seq Library Preparation Kit as per the manufacturer’s protocol and sequenced on an Illumina Next-Seq machine for 100 bp paired-end reads with the target of 50 million reads per sample.

Iso-Seq data were generated from brain, heart muscle, kidney, liver, lung, skeletal muscle, and placenta (cotyledon) tissue. Iso-Seq SMRT bell libraries were created according to the PacBio protocols. Briefly, two size-selected cDNA pools were created, one with an average cDNA size ~3 kb and the second with a cDNA size of ~7 kb. The two pools were then combined for SMRTbell™ Template Preparation. The final average library size was ~5 kb as measured by a bioanalyzer. Each SMRTbell library was loaded onto the Sequel at approximately 50 pM.

### Identification and phasing of full-length transcripts

The Iso-Seq data was processed using the isoseq3.1.0 software on the PacBio Bioconda (https://github.com/PacificBiosciences/pbbioconda). The process consists of (1) generating CCS reads, (2) classifying FLNC reads that have the 5‵, 3‵ cDNA primer sequence, and the polyA tail, (3) clustering FLNC reads at the isoform level and generating a draft consensus for each isoform, and (4) polishing each isoform to create HQ, full-length transcript sequences.

The HQ transcript sequences were then mapped to the Brahman reference genome using minimap2 (v2.15-r905) and filtered for alignments that had $\ge\!$99% coverage and $\ge \!$95% identity. Redundant and degraded transcripts were collapsed using the Cupcake tool (https://github.com/Magdoll/cDNA_Cupcake). SQANTI2^37^ was used to annotate transcripts that belong to seven distinct categories: (i) known isoforms with full-splice match, (ii) known isoforms with incomplete-splice match, (iii) novel isoforms in catalog, (iv) novel isoforms not in catalog, (v) antisense transcripts, (vi) transcripts that overlap with intergenic region, (vii) transcripts that overlap with genic regions.

In order to phase transcripts using the Iso-Seq data, we ran IsoPhase, which is a part of the Cupcake tool, against the Brahman reference. IsoPhase first piles up the FLNC reads of all the isoforms of a gene and calls substitution SNPs using a one-sided Fisher exact test with Bonferroni correction at a *P* value cut-off of 0.01. It then infers haplotypes based on the phasing information provided by the FLNC reads. The output defines the inferred haplotypes for each transcript and estimates the relative abundance of each allele. We ran IsoPhase using the pooled set of all FLNC reads from all tissues, then later demultiplex them to create an abundance matrix that is specific for each haplotype, per isoform FLNC count for each tissue. To compare the abundance of transcripts across tissues, we normalized the counts by dividing the FLNC counts for each haplotype isoform by the total number of FLNC counts in that tissue, multiplied by a million to obtain the transcript per million number.

### Mapping RNA-Seq and WGS reads from the F1 hybrid tissues

For RNA-Seq, read mapping was performed with Hisat2 v2.1.0^38^, whereas the genomic short reads were mapped using BWA v0.7.15^33^. SNPs were called using GATK v4^39^.

### Scaffold validation with recombination map

Scaffold contiguity was assessed using a previously published recombination map^40^. Briefly, the recombination map probe sequences were aligned using BWA MEM v0.7.15 to the scaffolds and the coordinates were arranged in a directed acyclic graph, using a custom script. A contiguity break between consecutive recombination map-ordered probes in the scaffolds was considered an error; however, we tolerated one mismatched probe in a window of three consecutive probes (Hamming distance = 1) to avoid false positive detection due to mapping ambiguity. Despite having Hi-C sequences, some scaffolds that belonged to chromosomes could not be joined together, which necessitated the use of recombination map markers to join and orientate these scaffolds.

### Gap filling and polishing

After checking scaffolds with recombination maps^40^, the Angus and Brahman scaffolds that contained 343 and 369 gaps, respectively, were gap filled with PBJelly^41^ v15.8.24 using haplotype-specific PacBio subreads. The default parameters of PBJelly were used, except for the support module, where the options captureOnly and spanOnly were used. This step closed 52 and 61 gaps in Angus and Brahman scaffolds, respectively. Two rounds of ArrowGrid (https://github.com/skoren/ArrowGrid) was run to polish the scaffolds to give quality scores.

### Assembly evaluation and genome annotation

The assemblies were evaluated with BUSCO v2.0.1^42^ and other metrics that include compression/expansion errors. Annotations were created using the Ensembl gene annotation system^43^ and the NCBI pipeline. Further detail on the annotation process is given in Supplementary Notes 3 and 4, and for assembly evaluation, detail is given in Supplementary Note 5.

### Repeat analysis

RepeatMasker version open-4.0.7 (http://www.repeatmasker.org) was used to search for repeats in the UOA_Angus_1 and UOA_Brahman_1 assemblies by identifying matches to RepBase (version RepBase23.10.embl)^44^. Repeats in the current water buffalo assembly (UOA_WB_1) and cattle assembly (UMD3.1.1) were downloaded from the NCBI. Repeats with matches ≤60% identity were filtered out. Centromeric repeats were identified by searching repeats that belonged to the family Satellite/centr in Repbase. We scored a sequence of repeat units as one block and counted the blocks, applying this method systematically throughout for all scaffolds. The vertebrate telomeric repeat, 6-mer TTAGGG, was identified by RepeatMasker. The search for at least 2 consecutive identical TTAGGG repeats within 1000 kb of chromosome ends was done to detect the presence of telomeres.

### Gap comparisons and sequence contiguity

To evaluate gaps and sequence contiguity, the Angus and Brahman assemblies were compared to the water buffalo, human, and Hereford cattle assemblies. Only sequences that belong to autosomes and sex chromosomes were retained for analysis, whereas unplaced and mitochondrial sequences were filtered out. The tool seqtk v1.2-r94 (https://github.com/lh3/seqtk) was used to count gaps with similar code implementation as those used for the water buffalo genome^9^.

### SNP and indel calls

Thirty-eight individuals with ~10× WGS short-read Illumina data representing 7 breeds were selected from the USMARC Beef Diversity Panel version 2.9 (MBCDPv2.9)^45^. The individuals selected for the panel were bulls with minimal pedigree relationships to maximize sampling of diverse alleles suitable for population genetics studies. The number of individuals per breed was as follow: six Angus, five Brahman, six Gelbvieh, six Hereford, five Red Angus, five Shorthorn, and five Simmental. These six taurine breeds were chosen on the basis that they were unlikely to carry *B. indicus* genetics given their history.

WGS data quality of each individual was checked with FASTQC v0.11.4^46^ and then trimmed with Trim Galore v0.4.2^47^ to a minimum length of 110 bp per read and Phred score of 20. Potential adapters in the sequence reads were removed using AdapterRemoval v2.2.1^48^. Following trimming, the reads were checked with FASTQC again to ensure that only HQ reads were retained. Reads were then mapped to both the Angus and Brahman assemblies separately using BWA v0.7.15^33^ with the option mem. Samtools v1.8^49^ was used to convert the resulting alignment to sorted bam format. Duplicate reads, which may be due to PCR artifacts, were marked with Picard v2.2.4^50^ MarkDuplicates. The bam files from each individual animal were merged with GATK v4^39^ MergeSamFiles function. Then the following series of GATK functions, AddOrReplaceReadGroups, HaplotypeCaller, CombineGVCFs, and GenotypeGVCFs, were applied to the alignment files to generate a variant call file in VCF v4.2 format. SNPs were filtered with VariantFiltration function using the parameters (QD < 2.0) || (FS > 60.0) || (MQ < 40.0) || (MQRankSum < -12.5) || (ReadPosRankSum < -8.0). Indels were filtered with VariantFiltration function using the parameters (QD < 2.0) || (FS > 200.0) || (ReadPosRankSum < -20.0). Annovar tool^51^ version dated 2017-07-17 was used to annotate the variants.

### SV and copy number variant analyses

WGS short-read data sets from the same 38 animals used for SNP and indel calls were aligned to the UOA_Angus_1, UOA_Brahman_1, and ARS-UCD1.2 with BWA MEM v0.7.15^33^ and further processed with Samtools v1.9^49^. Read-pair and split-read profile SVs were called with the lumpy-sv v0.2.13^52^ pipeline, lumpyexpress, using default parameters for each sample. lumpy-sv VCF files were converted to BEDPE format using the vcfToBedpe script included in the lumpy-sv software package. Copy number estimates for genomic segments were calculated from normalized WGS read depth using JaRMS v0.0.13 as previously described^53^. As JaRMS estimates of genomic copy number are distributed around a value of 1, as the normal diploid copy number count, we multiplied the level estimates from the JaRMS program by two to obtain the adjusted copy number state of genomic regions. JaRMS copy number estimates were used to estimate the population differentiation of taurine and indicine cattle on a per-gene basis using the *V*_st_ metric^12, 13^. A custom script (CalculateVstDifferences.py) was used to automate the calculation of *V*_st_ and generation of data tables for plotting. Genes that had a *V*_st_ > 0.3, which is equivalent to the top 1% *V*_st_, and a difference in average copy number between groups >3 were considered to have a significant difference in copy number between taurine and indicine populations.

In addition to using short WGS reads from the 38 individuals of 7 breeds to find SVs, the haplotype-resolved Angus and Brahman genomes were aligned with the HQ ARS-UCD1.2 cattle reference to assess SVs. The advantage of aligning to ARS-UCD1.2 was to standardize the SVs specific to each haplotype on a common coordinate system. Contigs obtained by breaking final scaffolds at gap positions from UOA_Angus_1 and UOA_Brahman_1 were aligned using nucmer v4^54^ to the ARS-UCD1.2 assembly to identify the larger structural differences (50–10,000 bp) using Assemblytics^11^. The nucmer alignment parameters were –maxmatch -t 4 -l 100 -c 500, which was followed by delta-filter with the option -g. Assemblytics parameters followed the default settings, which were “Unique sequence length required: 10000, Maximum variant size: 10000, Minimum variant size: 50.” The overlap of SVs with Ensembl annotation of Hereford cattle ARS-UCD1.2 release 96 were identified with GenomicFeatures and systemPipeR R packages.

### Identification, copy number and phylogenetic tree of FADS2P1

All chromosomes from Brahman were aligned to the corresponding Angus chromosomes using the dot plot tool Gepard v1.4^55^. Genomic regions that differed between the two subspecies were isolated for further scrutiny. Of all the regions analyzed, one particular locus on Brahman chromosome 15 at position ~4 Mb covering ~200 kb diverged from the corresponding Angus chromosome. Further analysis revealed an extra copy of *FADS2P1* in the Brahman genome. BLASTP^56^ analysis identified two copies of *FADS2P1* in Angus, Hereford, water buffalo, and goat, whereas only Brahman had three copies of this gene. A maximum likelihood tree with 1000 bootstraps was constructed for *FADS2P1* homologs using RAxML v8^57^ with substitution model PROTGAMMAAUTO. The conservation of synteny around the FADS2P1 locus was investigated by alignments of Angus to Brahman and Angus to Hereford using nucmer v4^54^ and displayed with Ribbon^58^.

### Positive selection analysis on *FADS2P1*

The observation of an indicus-specific *FADS2P1* residing in a divergent region prompted further investigation into the possibility that the gene is under positive selection. Homologs of *FADS2P1* in Brahman, Angus, and water buffalo were subjected to CODEML analysis as implemented in PAML v4.8^59^. Selective pressure acting on a gene can be estimated by the rate ratio (*ω*) of non-synonymous (amino acid changes) to synonymous (silent changes) substitutions. Detection of *ω* > 1 is a sign of positive selection, and the site models, namely, M7 and M8 in PAML, which allow *ω* to vary among sites, were used to detect positive selection. Protein sequences of *FADS2P1* homologs were aligned using Muscle^60^, and the corresponding nucleotides were mapped back onto the amino acid alignment using PAL2NAL^61^ with gap removal. The tree topology used to run CODEML was a maximum likelihood gene tree calculated from RAxML^57^. Model M8 was compared with M7 using the likelihood ratio test (LRT) to evaluate if the model with positive selection was favored. More detail on similar positive selection methodology can be found in our study on mammalian glutathione S-transferases^62^.

### Statistical analysis

R/Bioconductor was used for all statistical analyses. Significance of positively selected sites found in *FADS2P1* were evaluated using the LRT, with the test statistic *t*_LR_ = 2[*l*(Model 8) − *l*(Model 7)].

### Reporting summary

Further information on research design is available in the Nature Research Reporting Summary linked to this article.

## Supplementary information

Supplementary Information

Reporting Summary

## Data Availability

The PacBio reads, Hi-C reads, RNA-Seq, Iso-Seq, and Illumina paired-end reads are available in the SRA under BioProject PRJNA432857. The 38 individuals from seven breeds used for variant calls were downloaded from the BioProject PRJNA324822. The assemblies ARS-UCD1.2 (GCF_002263795.1), Bos_taurus_UMD_3.1.1 (GCF_000003055.6), ARS1 (GCF_001704415.1), and UOA_WB_1 (GCF_003121395.1) were downloaded from the NCBI. Intermediary assembly FASTA files and other miscellaneous information are available from the corresponding authors upon request. Annotation files of UOA_Angus_1 and UOA_Brahman_1 are available through Ensembl. Primary accessions number: BioProject: PRJNA432857; GenBank assembly accession for UOA_Angus_1: GCA_003369685.2; and GenBank assembly accession for UOA_Brahman_1: GCA_003369695.2.

## Code availability

Custom scripts can be found at GitHub repository at the following URL: https://github.com/lloydlow/BrahmanAngusAssemblyScripts.
