# Supplementary material for: Haplotype-resolved genomes provide insights into structural variation and gene content in Angus and Brahman cattle
Source: Nat Commun. 2020 Apr 29;11:2071. doi: 10.1038/s41467-020-15848-y (PMC7190621; doi:10.1038/s41467-020-15848-y)
Supplement: Supplementary file 1 — Supplementary Information [file 41467_2020_15848_MOESM1_ESM.pdf]

Supplementary Figures

Supplementary Figure 1: **Comparison of chromosome sizes between Angus, Brahman and Hereford assemblies.**

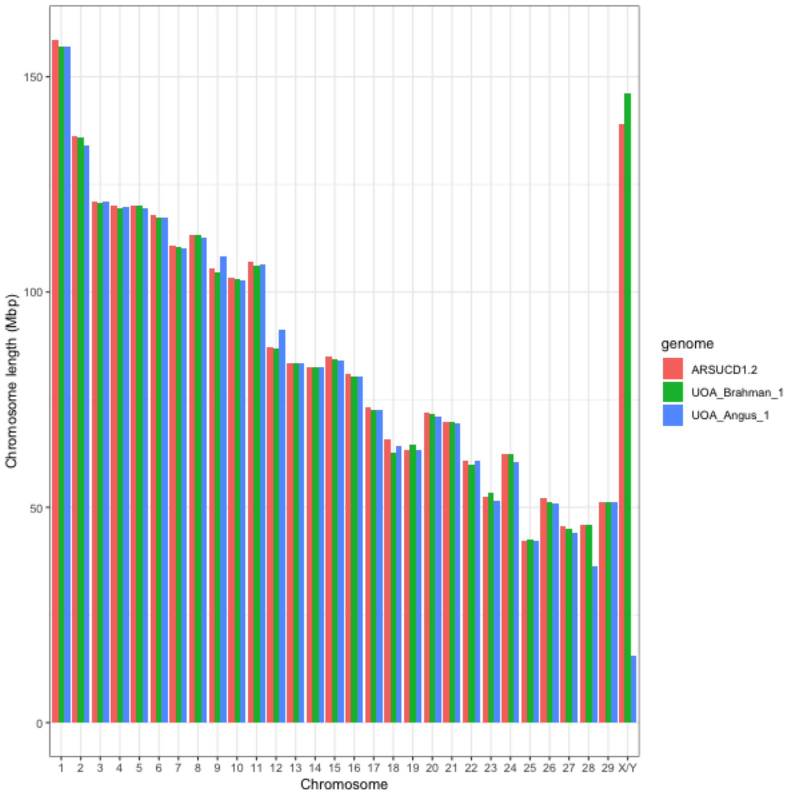

Supplementary Figure 2: **Distribution of un-gapped contig lengths in the three cattle breeds (UOA_Angus_1, UOA_Brahman_1 and ARS-UCD1.2) and water buffalo (UOA_WB_1).** Dot plots of individual values are overlaid on top of boxplots to show minima and maxima as circles. The bounds of box show the 25th and 75th percentile, with the median drawn as a thick line between these two quartiles.

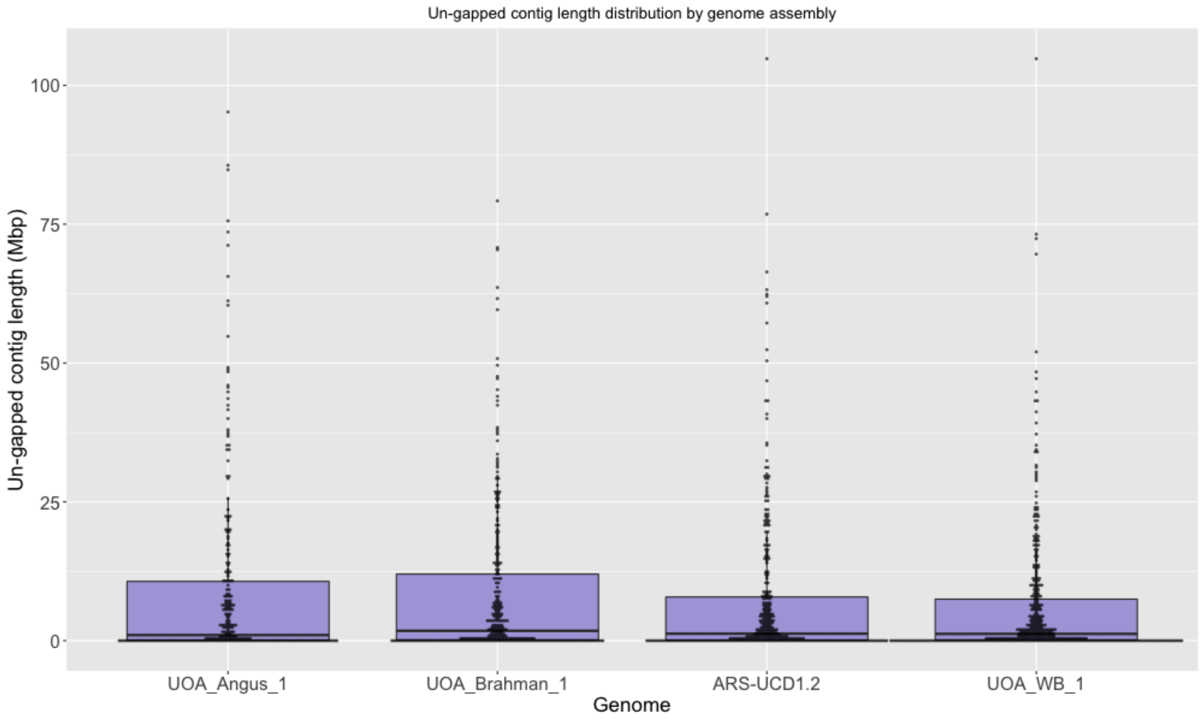

Supplementary Figure 3: **The count in (log$_{10}$ scale) of LINE/L1, LINE/RTE-BovB and Satellite/centromeric repeats in cattle genome assemblies.** The count for Satellite/centromeric is of a sequence of repeat units.

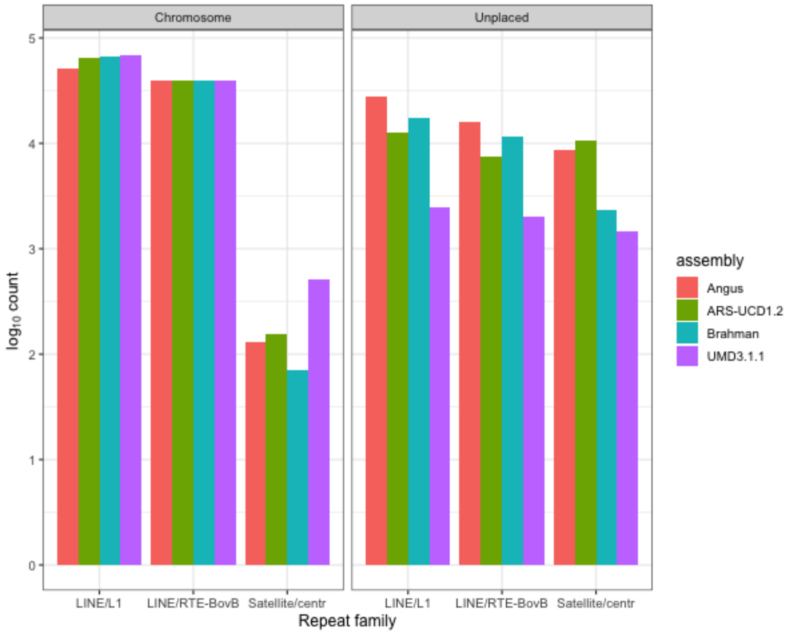

Supplementary Figure 4: **Coverage plot of *FADS2P1* in individuals of different cattle breeds.** The dashed blue line indicates the expected haploid coverage. As *FADS2P1* is member of a gene family, short reads that belong to other gene family members could potentially have mis-mapped to this region, which explains the non-zero coverage in taurine breeds at certain positions across the gene.

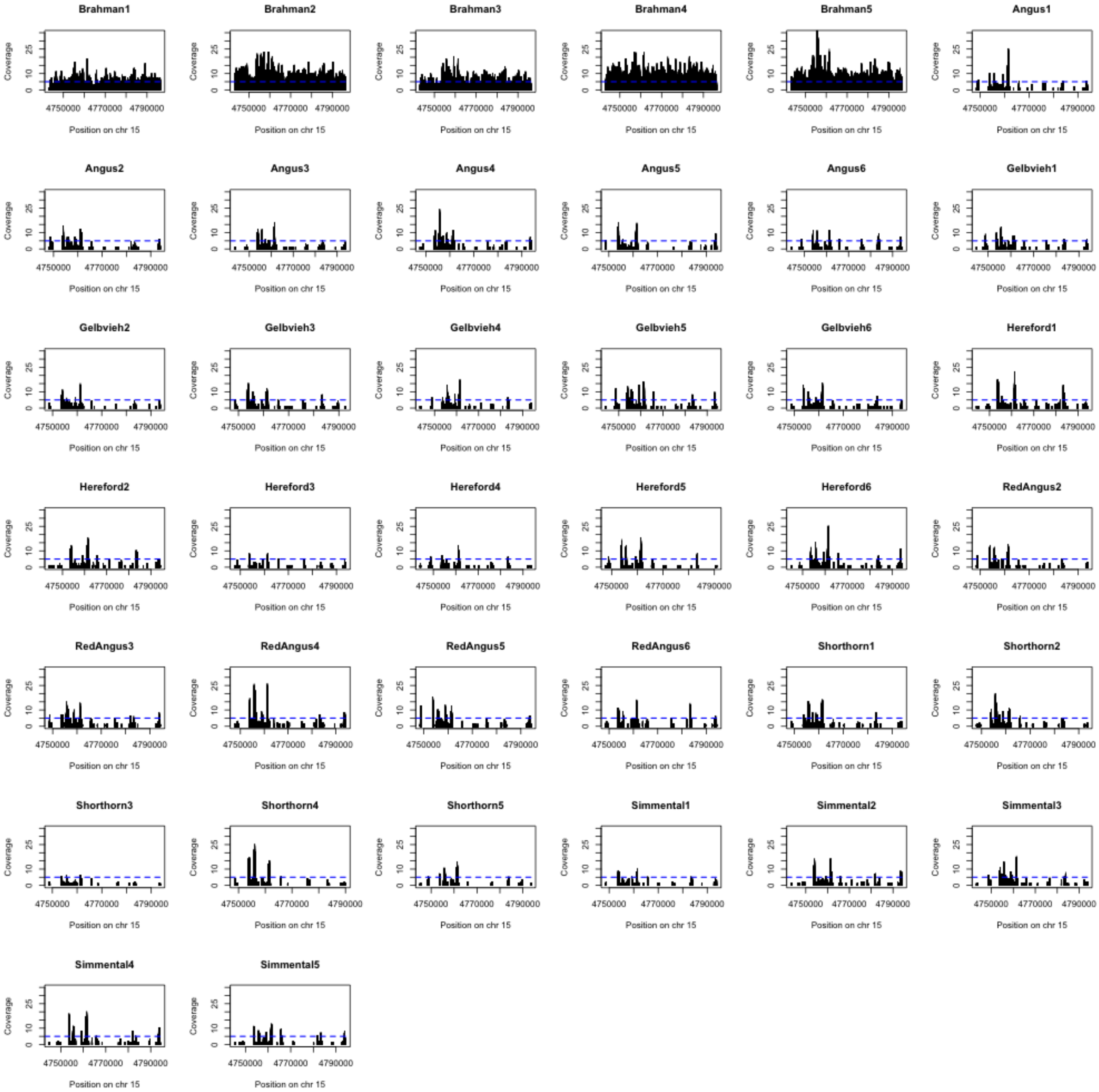

Supplementary Figure 5: **Distribution and breed specificity of Brahman and Angus structural variants.** a) Count of structural variants (SVs) categorized as deletion, insertion, repeat contraction, repeat expansion, tandem contraction, and tandem expansion by Assemblytics. The Hereford ARS-UCD1.2 was used as the common reference to call SVs in both Brahman and Angus contigs. b) Venn diagrams showing overlap of six classes of SVs between Brahman and Angus.

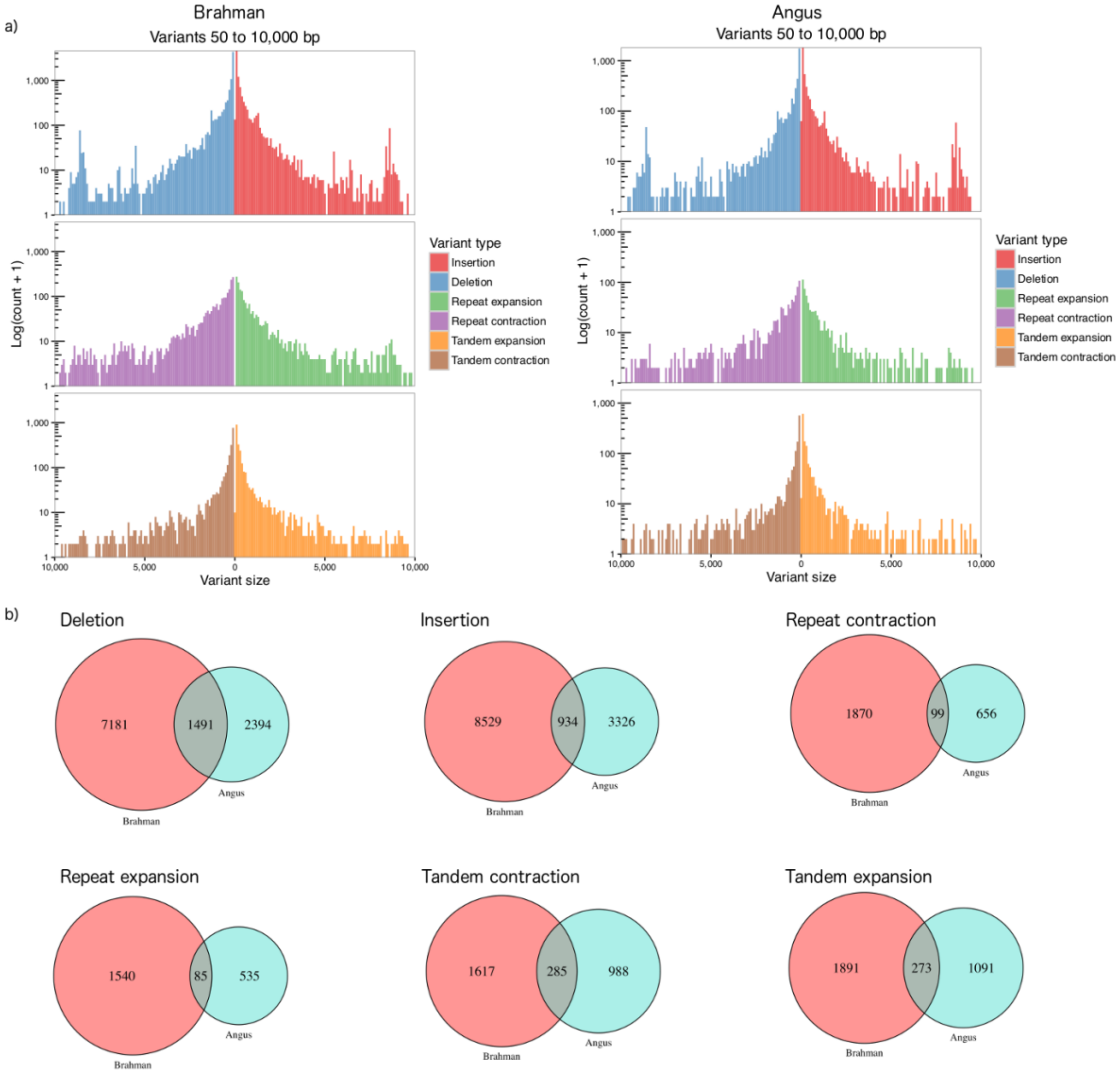

Supplementary Figure 6: **Analysis of copy number variations using different reference assemblies.** Population differentiation for copy number variations (CNV) as estimated by V_{ST} along each chromosome for the taurine and indicine comparison using a) UOA_Angus_1 and b) ARS-UCD1.2 as the reference genome.

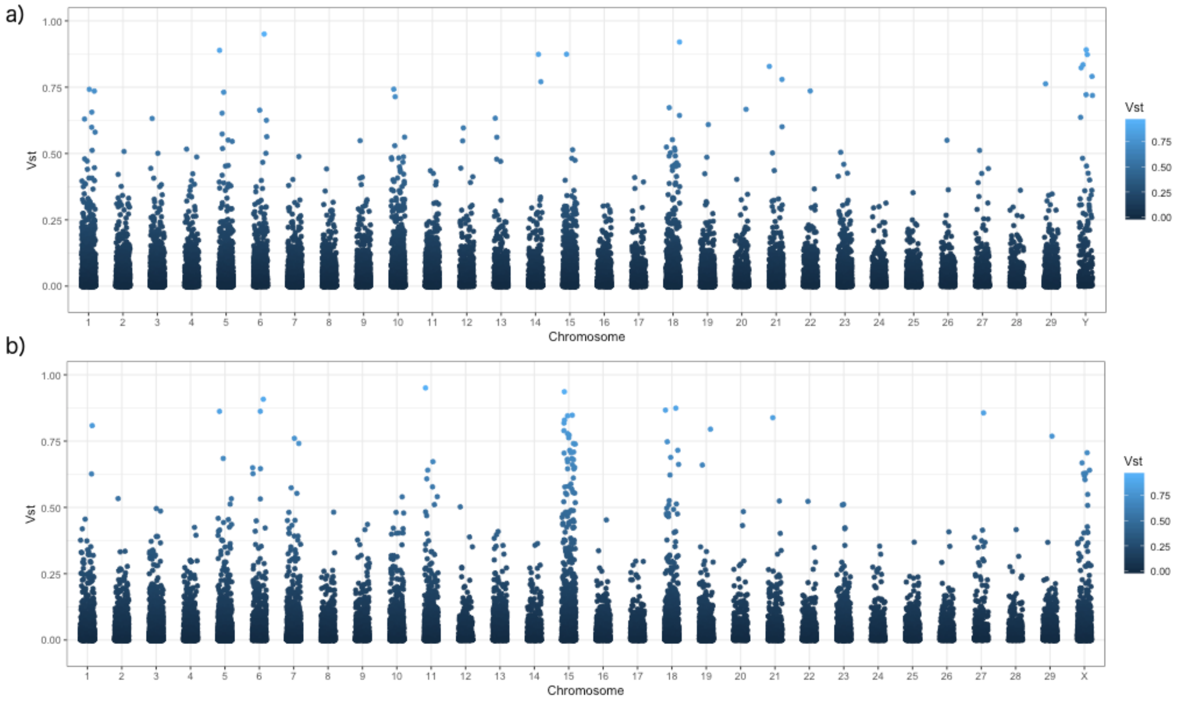

Supplementary Figure 7: **Full-length Iso-Seq transcripts (bottom) and RNA-Seq coverage for *ARIH2* in the brain tissue (top).**

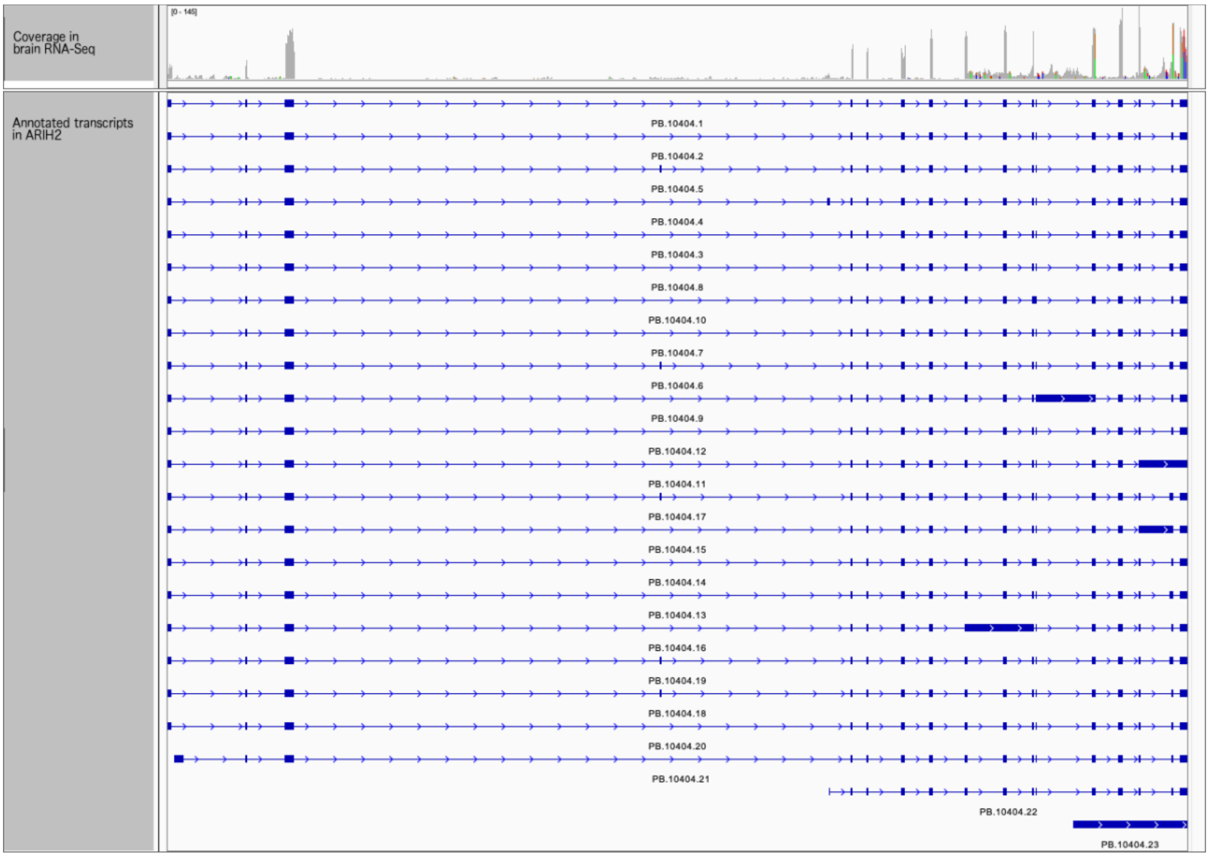

Supplementary Figure 8: **Normalized tissue-specific transcript counts for genes with allelic imbalance and higher expression of the Brahman allele in brain.** a) Calmodulin. b) Pregnancy-associated glycoprotein 1 (*PAG1*).

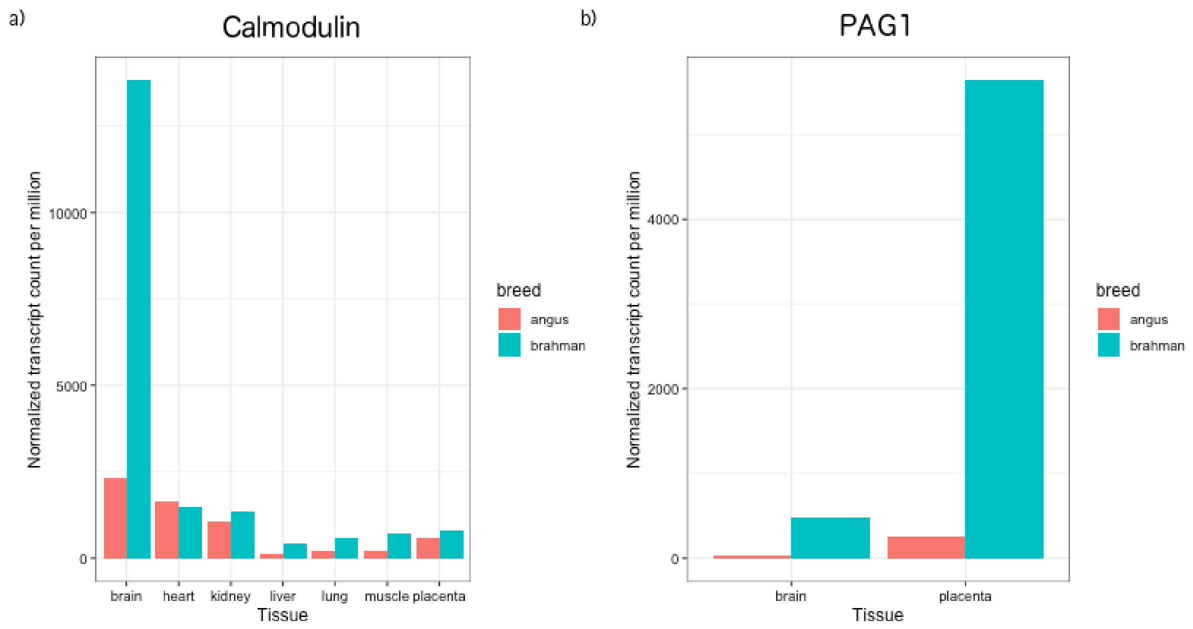

Supplementary Figure 9: **Distribution of unassigned PacBio WGS read length.** For each boxplot, the minimum is represented as the end point of the vertical line extending downward from the box whereas the maximum is the highest value shown as a circle. The bounds of box show the 25th and 75th percentile, with the median drawn as a thick line between these two quartiles.

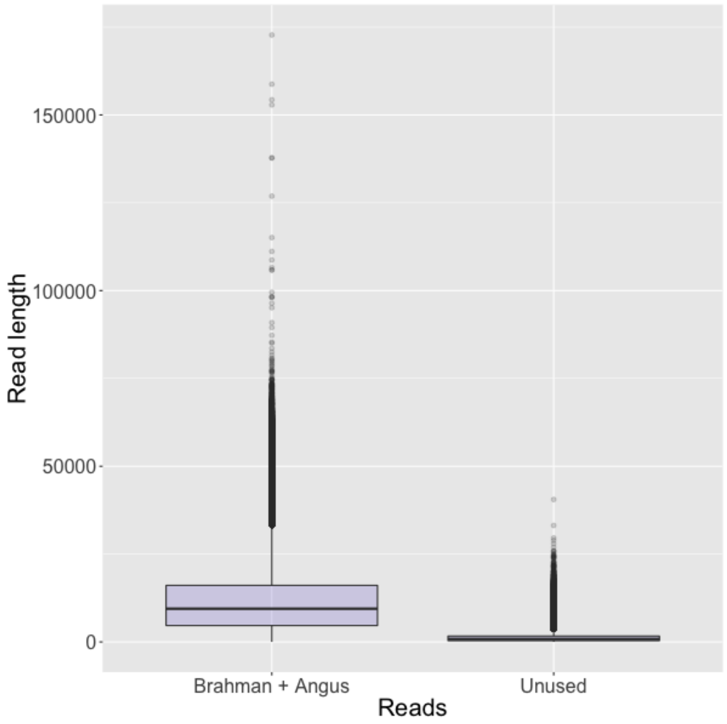

Supplementary Tables

Supplementary Table 1: **Annotation features in Brahman, Angus and Hereford assemblies.** Comparison of UOA_Brahman_1, UOA_Angus_1, and ARS-UCD1.2 features annotated by the EMBL-EBI pipeline release 96. The columns with X and Y next to each breed show the annotated features in sex chromosomes of the corresponding assemblies.

Feature	Brahman autosomes	Angus autosomes	Brahman X	Angus Y
gene	28547	28758	1363	192
lncRNA	3224	3269	153	25
miRNA	776	867	71	1
misc RNA	361	359	25	0
processed pseudogene	89	95	13	2
protein coding	21170	21266	948	153
pseudogene	458	451	38	3
rRNA	411	392	13	2
ribozyme	7	6	0	0
sRNA	3	3	0	0
scaRNA	32	31	1	0
snRNA	1130	1141	65	5
snoRNA	748	751	36	1

Supplementary Table 2: **Assembly quality score values.**

Statistic	Description	Angus	Brahman
QV	Quality value	44.63	46.38
COMPR_PE	Low CE-statistics computed on PE reads	211314	211783
STRECH_PE	High CE-statistics computed on MP reads	106768	92494
LOW_COV_PE	Low read coverage areas	61490	81218
LOW_NORM_COV_PE	Low paired-read coverage areas	58223	79034
HIGH_COV_PE	High read coverage areas	4329	3920
HIGH_NORM_COV_PE	High paired-read coverage areas	3803	2775
HIGH_SPAN_PE	High number of PE reads with pair mapped in a different scaffold	1808	2464
HIGH_SINGLE_PE	High number of PE reads with unmapped pair	30	96
HIGH_OUTIE_PE	High number of mis-oriented or too distant PE reads	15	10

Note: CE, compression/expansion; PE, paired-end

Supplementary Table 3: **BUSCO assessment of the completeness of single-copy orthologs for Angus and Brahman genomes.**

Description	Angus	Brahman
Complete BUSCOs	3813	3839
Complete and single-copy BUSCOs	3764	3790
Complete and duplicated BUSCOs	49	49
Fragmented BUSCOs	130	123
Missing BUSCOs	161	142
Total BUSCO groups searched	4104	4104
BUSCO completeness (%)	92.9	93.5

Supplementary Table 4: **Site models of CODEML for *FADS2P1* and positively selected sites.**

Model	Log-likelihood	2Δ(In L)	P-value[b]	Positively selected sites[a]	Tree length	Average dN	Average dS
M7	-3149.50						
M8	-3132.38	17.12	0.00019	237V<br>271V<br>294H<br>305C<br>306T<br>307V**<br>311L<br>312F<br>315V<br>317L<br>324A<br>327C**<br>328R<br>329R<br>330S<br>370P	0.5777	0.0168	0.0193

[a]Bayes Empirical Bayes (BEB) was used to calculate posterior probabilities and only those with $Prob(\omega > 1) > 0.95$ are shown. ** indicates those with $Prob(\omega > 1) > 0.99$. Amino acid position follows Brahman ENSBIXP00005018486.1, which is the indicus-specific copy of *FADS2P1*. [b]P-value from likelihood ratio test.

Supplementary Table 5: **Genes identified in the selective sweep intervals.**

chr	start	end	Ensembl ID	Name	biotype	Indicine mean proportion alternate allele	Taurine mean proportion alternate allele
1	3500000	3600000	ENSBIXG00005005115	SCAF4	protein coding	0.048	0.401
1	81300000	81400000	ENSBIXG00005003979	SENP2	protein coding	0.093	0.405
1	81300000	81400000	ENSBIXG00005023240	LIPH	protein coding	0.093	0.405
1	81300000	81400000	ENSBIXG00005023174	RF00001	rRNA	0.093	0.405
1	107100000	107200000	ENSBIXG00005012541	not available	protein coding	0.100	0.584
1	136400000	136500000	ENSBIXG00005025046	ACAD11	protein coding	0.073	0.482
1	136400000	136500000	ENSBIXG00005024967	DNAJC13	protein coding	0.073	0.482
2	24300000	24400000	ENSBIXG00005005621	HAT1	protein coding	0.092	0.410
2	24300000	24400000	ENSBIXG00005027305	not available	protein coding	0.092	0.410
2	24300000	24400000	ENSBIXG00005027277	SLC25A12	protein coding	0.092	0.410
3	600000	700000	ENSBIXG00005024597	GPR161	protein coding	0.100	0.483
3	600000	700000	ENSBIXG00005024283	DCAF6	protein coding	0.100	0.483
3	600000	700000	ENSBIXG00005004103	RF00201	snoRNA	0.100	0.483
3	600000	700000	ENSBIXG00005023946	not available	protein coding	0.100	0.483
3	53800000	53900000	ENSBIXG00005030064	LRRC8C	protein coding	0.100	0.447
3	53800000	53900000	ENSBIXG00005030089	LRRC8B	protein coding	0.100	0.447
4	400000	500000	ENSBIXG00005018118	ESYT2	protein coding	0.092	0.563
4	400000	500000	ENSBIXG00005018040	NCAPG2	protein coding	0.092	0.563
4	1000000	1100000	ENSBIXG00005002902	not available	protein coding	0.064	0.459
4	52900000	53000000	ENSBIXG00005014470	not available	protein coding	0.043	0.409
4	72600000	72700000	ENSBIXG00005028158	CDHR3	protein coding	0.020	0.548
4	72600000	72700000	ENSBIXG00005004834	not available	miRNA	0.020	0.548
4	95100000	95200000	ENSBIXG00005018443	CRPPA	protein coding	0.083	0.474
5	42800000	42900000	ENSBIXG00005004767	FGD4	protein coding	0.070	0.537
5	42800000	42900000	ENSBIXG00005027380	RF00026	snRNA	0.070	0.537
5	48900000	49000000	ENSBIXG00005023898	SYN3	protein coding	0.091	0.591
5	106500000	106600000	ENSBIXG00005000567	not available	protein coding	0.100	0.518
6	116700000	116800000	ENSBIXG00005005681	not available	protein coding	0.070	0.409
6	116700000	116800000	ENSBIXG00005008386	not available	lncRNA	0.070	0.409
6	116700000	116800000	ENSBIXG00005008383	not available	protein coding	0.070	0.409
6	116700000	116800000	ENSBIXG00005000173	not available	lncRNA	0.070	0.409
6	116700000	116800000	ENSBIXG00005008365	FGFRL1	protein coding	0.070	0.409
6	116700000	116800000	ENSBIXG00005008347	not available	lncRNA	0.070	0.409
6	116700000	116800000	ENSBIXG00005008330	IDUA	protein coding	0.070	0.409
7	50000000	50100000	ENSBIXG00005019655	not available	lncRNA	0.040	0.437

**7**	50000000	50100000	ENSBIXG00005003205	IL17B	protein coding	0.040	0.437
**7**	50000000	50100000	ENSBIXG00005003199	PCYOX1L	protein coding	0.040	0.437
**7**	50000000	50100000	ENSBIXG00005019586	not available	protein coding	0.040	0.437
**7**	50000000	50100000	ENSBIXG00005003178	AFAP1L1	protein coding	0.040	0.437
**7**	50000000	50100000	ENSBIXG00005019455	not available	protein coding	0.040	0.437
**7**	51900000	52000000	ENSBIXG00005003009	JAKMIP2	protein coding	0.100	0.405
**7**	66100000	66200000	ENSBIXG00005000684	HSPA4	protein coding	0.100	0.459
**7**	81100000	81200000	ENSBIXG00005020813	not available	lncRNA	0.085	0.470
**8**	7300000	7400000	ENSBIXG00005029063	DEFB136	protein coding	0.014	0.414
**8**	7300000	7400000	ENSBIXG00005029055	CTSB	protein coding	0.014	0.414
**8**	7300000	7400000	ENSBIXG00005029005	FDFT1	protein coding	0.014	0.414
**8**	10800000	10900000	ENSBIXG00005027580	not available	protein coding	0.100	0.431
**8**	10800000	10900000	ENSBIXG00005027560	ESCO2	protein coding	0.100	0.431
**8**	10800000	10900000	ENSBIXG00005004734	CCDC25	protein coding	0.100	0.431
**8**	52900000	53000000	ENSBIXG00005006324	VPS13A	protein coding	0.040	0.495
**8**	57500000	57600000	ENSBIXG00005011007	not available	protein coding	0.055	0.730
**8**	85000000	85100000	ENSBIXG00005000168	not available	pseudogene	0.053	0.433
**9**	32400000	32500000	ENSBIXG00005010729	CEP85L	protein coding	0.086	0.447
**9**	40600000	40700000	ENSBIXG00005008671	FIG4	protein coding	0.044	0.468
**9**	40600000	40700000	ENSBIXG00005008574	AK9	protein coding	0.044	0.468
**9**	85800000	85900000	ENSBIXG00005003851	SASH1	protein coding	0.083	0.475
**10**	31600000	31700000	ENSBIXG00005004621	not available	protein coding	0.092	0.501
**10**	31600000	31700000	ENSBIXG00005004584	CAPN3	protein coding	0.092	0.501
**10**	72600000	72700000	ENSBIXG00005007972	GPHN	protein coding	0.033	0.458
**10**	102100000	102200000	ENSBIXG00005017943	TTC7B	protein coding	0.100	0.478
**10**	102100000	102200000	ENSBIXG00005002910	RF00614	snoRNA	0.100	0.478
**11**	37900000	38000000	ENSBIXG00005017832	CFAP36	protein coding	0.100	0.498
**11**	37900000	38000000	ENSBIXG00005017770	PPP4R3B	protein coding	0.100	0.498
**11**	37900000	38000000	ENSBIXG00005017706	PNPT1	protein coding	0.100	0.498
**11**	45700000	45800000	ENSBIXG00005001920	NCK2	protein coding	0.075	0.406
**11**	45700000	45800000	ENSBIXG00005001916	not available	lncRNA	0.075	0.406
**11**	45700000	45800000	ENSBIXG00005014366	not available	lncRNA	0.075	0.406
**11**	45700000	45800000	ENSBIXG00005014363	RF00619	snRNA	0.075	0.406
**11**	45700000	45800000	ENSBIXG00005006127	not available	lncRNA	0.075	0.406
**11**	45800000	45900000	ENSBIXG00005007270	TTL	protein coding	0.100	0.438
**13**	59700000	59800000	ENSBIXG00005022377	PIP4K2A	protein coding	0.060	0.441
**14**	46600000	46700000	ENSBIXG00005016564	EXT1	protein coding	0.087	0.432
**14**	47000000	47100000	ENSBIXG00005007032	MED30	protein coding	0.022	0.461
**14**	64800000	64900000	ENSBIXG00005011378	VPS13B	protein coding	0.092	0.413
**14**	64800000	64900000	ENSBIXG00005011322	RF00156	snoRNA	0.092	0.413
**15**	68600000	68700000	ENSBIXG00005002944	AMOTL1	protein coding	0.085	0.499
**16**	4300000	4400000	ENSBIXG00005010567	MAPKAPK2	protein coding	0.085	0.473

**16**	4300000	4400000	ENSBIXG00005010507	not available	protein coding	0.085	0.473
**16**	36100000	36200000	ENSBIXG00005023955	ATP1B1	protein coding	0.082	0.495
**16**	36100000	36200000	ENSBIXG00005023904	RF00155	snoRNA	0.082	0.495
**16**	36100000	36200000	ENSBIXG00005023880	NME7	protein coding	0.082	0.495
**16**	36100000	36200000	ENSBIXG00005004083	not available	protein coding	0.082	0.495
**16**	48600000	48700000	ENSBIXG00005016454	not available	protein coding	0.073	0.543
**16**	48600000	48700000	ENSBIXG00005002621	DFFB	protein coding	0.073	0.543
**16**	48600000	48700000	ENSBIXG00005016419	CEP104	protein coding	0.073	0.543
**16**	48600000	48700000	ENSBIXG00005002608	not available	miRNA	0.073	0.543
**16**	48600000	48700000	ENSBIXG00005016351	LRRC47	protein coding	0.073	0.543
**16**	48600000	48700000	ENSBIXG00005016324	SMIM1	protein coding	0.073	0.543
**16**	48600000	48700000	ENSBIXG00005016305	not available	protein coding	0.073	0.543
**16**	54600000	54700000	ENSBIXG00005008560	RC3H1	protein coding	0.091	0.430
**16**	54800000	54900000	ENSBIXG00005000199	RABGAP1L	protein coding	0.035	0.447
**16**	79400000	79500000	ENSBIXG00005019828	PPP1R12B	protein coding	0.050	0.423
**16**	79400000	79500000	ENSBIXG00005019795	RF00004	snRNA	0.050	0.423
**19**	27100000	27200000	ENSBIXG00005010360	not available	protein coding	0.092	0.403
**19**	27100000	27200000	ENSBIXG00005010357	MIS12	protein coding	0.092	0.403
**19**	27100000	27200000	ENSBIXG00005010333	DERL2	protein coding	0.092	0.403
**19**	27100000	27200000	ENSBIXG00005010287	DHX33	protein coding	0.092	0.403
**19**	27100000	27200000	ENSBIXG00005010261	NUP88	protein coding	0.092	0.403
**19**	27100000	27200000	ENSBIXG00005010218	RPAIN	protein coding	0.092	0.403
**19**	27100000	27200000	ENSBIXG00005001571	RABEP1	protein coding	0.092	0.403
**19**	46800000	46900000	ENSBIXG00005022992	CRHR1	protein coding	0.075	0.468
**20**	18500000	18600000	ENSBIXG00005013524	not available	protein coding	0.050	0.444
**20**	18500000	18600000	ENSBIXG00005013520	RF02160	misc RNA	0.050	0.444
**20**	18500000	18600000	ENSBIXG00005013509	RF02159	misc RNA	0.050	0.444
**21**	7800000	7900000	ENSBIXG00005023285	not available	lncRNA	0.090	0.401
**21**	7800000	7900000	ENSBIXG00005023277	not available	lncRNA	0.090	0.401
**21**	7800000	7900000	ENSBIXG00005023256	IGF1R	protein coding	0.090	0.401
**21**	49000000	49100000	ENSBIXG00005025684	SEC23A	protein coding	0.056	0.571
**21**	49000000	49100000	ENSBIXG00005004365	GEMIN2	protein coding	0.056	0.571
**21**	49000000	49100000	ENSBIXG00005025542	not available	protein coding	0.056	0.571
**21**	49000000	49100000	ENSBIXG00005004347	TRAPPC6B	protein coding	0.056	0.571
**21**	49000000	49100000	ENSBIXG00005025501	PNN	protein coding	0.056	0.571
**23**	14700000	14800000	ENSBIXG00005010033	KIF6	protein coding	0.094	0.488
**23**	43400000	43500000	ENSBIXG00005028562	not available	lncRNA	0.083	0.573
**24**	200000	300000	ENSBIXG00005023903	RF00001	rRNA	0.055	0.702
**24**	500000	600000	ENSBIXG00005006840	PARD6G	protein coding	0.064	0.544
**24**	600000	700000	ENSBIXG00005004059	ADNP2	protein coding	0.073	0.587
**24**	600000	700000	ENSBIXG00005023670	not available	protein coding	0.073	0.587
**24**	600000	700000	ENSBIXG00005023663	RBFA	protein coding	0.073	0.587

**24**	37200000	37300000	ENSBIXG00005013581	SMCHD1	protein coding	0.033	0.507
**24**	37200000	37300000	ENSBIXG00005013554	EMILIN2	protein coding	0.033	0.507
**24**	37200000	37300000	ENSBIXG00005013545	RF00026	snRNA	0.033	0.507
**27**	41300000	41400000	ENSBIXG00005026717	THRB	protein coding	0.017	0.574
**27**	41300000	41400000	ENSBIXG00005026549	NR1D2	protein coding	0.017	0.574
**28**	18800000	18900000	ENSBIXG00005020285	not available	protein coding	0.027	0.415
**28**	18800000	18900000	ENSBIXG00005020283	ADO	protein coding	0.027	0.415
**28**	18800000	18900000	ENSBIXG00005020273	EGR2	protein coding	0.027	0.415
**29**	19200000	19300000	ENSBIXG00005028250	ETS1	protein coding	0.100	0.458

Supplementary Table 6: **Annotation of SNP and INDEL variants.** Short read data from either Angus or Brahman was mapped to the corresponding reference genomes. After GATK variant calling and filtering of variants, Annovar was used to annotate the variants identified. Note: SNV is single nucleotide variant.

Description	Angus	Brahman
number of animals	6	5
nonsynonymous SNV	53730	79170
stop gain	871	1253
stop loss	220	267
synonymous SNV	47843	96675
frameshift deletion	1350	1866
frameshift insertion	1120	1397
nonframeshift deletion	519	845
nonframeshift insertion	386	588
stop gain	61	101
stop loss	9	15

Supplementary Table 7: **Breed-specific structural variant (SV) type and over/under-represented gene ontology for biological processes.** The null hypothesis is all cattle genes and genes that overlapped with SV intervals are equally likely to be found in a particular GO-Slim Biological Process. We have used two-sided Fisher's Exact test and applied correction to the p-value using False Discovery Rate (FDR). The raw p-values are from Fisher's Exact test before FDR adjustment.

**Angus-specific insertion SV**				
PANTHER GO-Slim Biological Process	Over/Under-represented GO	Fold enrichment	Raw P-value	FDR
cellular response to stimulus (GO:0051716)	+	1.75	8.49E-06	1.52E-02
**Angus-specific tandem contraction SV**				
PANTHER GO-Slim Biological Process	Over/Under-represented GO	Fold enrichment	Raw P-value	FDR
synaptic vesicle endocytosis (GO:0048488)	+	24.92	3.75E-06	3.35E-03
synaptic vesicle cycle (GO:0099504)	+	19.29	1.14E-05	5.08E-03
organophosphate biosynthetic process (GO:0090407)	+	15.43	1.96E-04	3.18E-02
peptide metabolic process (GO:0006518)	+	13	6.40E-05	1.91E-02
regulation of cation transmembrane transport (GO:1904062)	+	12.46	7.71E-05	1.97E-02
regulation of ion transmembrane transport (GO:0034765)	+	12.46	7.71E-05	1.72E-02
regulation of ion transport (GO:0043269)	+	10.47	8.03E-06	4.78E-03
regulation of transport (GO:0051049)	+	7.9	4.43E-05	1.58E-02
membrane invagination (GO:0010324)	+	6.2	1.86E-04	3.70E-02
vesicle budding from membrane (GO:0006900)	+	6.2	1.86E-04	3.33E-02
regulation of localization (GO:0032879)	+	6.06	3.37E-06	6.03E-03
**Brahman-specific insertion SV**				
PANTHER GO-Slim Biological Process	Over/Under-represented GO	Fold enrichment	Raw P-value	FDR
release of sequestered calcium ion into cytosol (GO:0051209)	+	6.93	1.87E-04	2.78E-02
negative regulation of sequestering of calcium ion (GO:0051283)	+	6.67	2.29E-04	2.56E-02
phospholipid translocation (GO:0045332)	+	5.71	8.27E-05	2.11E-02
organophosphate biosynthetic process (GO:0090407)	+	5.59	5.72E-04	4.09E-02
lipid translocation (GO:0034204)	+	5.57	9.78E-05	2.19E-02
sequestering of calcium ion (GO:0051208)	+	5.57	9.78E-05	1.94E-02
positive regulation of cell migration (GO:0030335)	+	5.5	2.56E-04	2.54E-02
regulation of ion transport (GO:0043269)	+	3.71	2.28E-04	2.71E-02

lipid transport (GO:0006869)	+	3.32	3.42E-04	2.91E-02
negative regulation of cellular process (GO:0048523)	+	3.27	2.35E-04	2.47E-02
microtubule-based movement (GO:0007018)	+	3.19	7.74E-04	4.77E-02
phosphate-containing compound metabolic process (GO:0006796)	+	3.12	5.74E-04	3.94E-02
regulation of transport (GO:0051049)	+	3.04	7.32E-04	4.85E-02
lipid localization (GO:0010876)	+	3.04	7.32E-04	4.67E-02
regulation of membrane potential (GO:0042391)	+	2.96	1.56E-04	2.54E-02
organophosphate metabolic process (GO:0019637)	+	2.88	2.11E-04	2.90E-02
regulation of localization (GO:0032879)	+	2.74	2.50E-05	7.44E-03
membrane fusion (GO:0061025)	+	2.7	4.27E-04	3.18E-02
microtubule-based process (GO:0007017)	+	2.61	1.04E-04	1.86E-02
macromolecule localization (GO:0033036)	+	2.53	4.23E-04	3.29E-02
signal transduction (GO:0007165)	+	1.65	2.29E-07	1.36E-04
intracellular signal transduction (GO:0035556)	+	1.64	3.88E-04	3.15E-02
cellular response to stimulus (GO:0051716)	+	1.57	6.15E-07	2.20E-04
localization (GO:0051179)	+	1.41	2.21E-04	2.83E-02
cellular process (GO:0009987)	+	1.27	4.24E-07	1.90E-04
gene expression (GO:0010467)	-	0.62	3.32E-04	2.97E-02
sensory perception (GO:0007600)	-	0.35	2.75E-04	2.59E-02
sensory perception of chemical stimulus (GO:0007606)	-	0.05	4.59E-08	4.10E-05
detection of chemical stimulus involved in sensory perception (GO:0050907)	-	< 0.01	1.12E-08	2.00E-05
**Brahman-specific deletion SV**				
PANTHER GO-Slim Biological Process	Over/Under-represented GO	Fold enrichment	Raw P-value	FDR
organophosphate biosynthetic process (GO:0090407)	+	5.9	4.21E-04	4.18E-02
small molecule biosynthetic process (GO:0044283)	+	5.5	2.49E-04	3.43E-02
sequestering of calcium ion (GO:0051208)	+	5.22	3.38E-04	4.32E-02
calcium-mediated signaling (GO:0019722)	+	3.82	1.75E-04	2.84E-02
cellular calcium ion homeostasis (GO:0006874)	+	3.22	8.44E-06	5.03E-03
calcium ion homeostasis (GO:0055074)	+	3.21	9.16E-06	4.09E-03
negative regulation of cellular process (GO:0048523)	+	3.2	4.55E-04	4.28E-02
divalent inorganic cation homeostasis (GO:0072507)	+	2.98	1.64E-05	5.86E-03
ion homeostasis (GO:0050801)	+	2.43	5.87E-05	1.31E-02
inorganic ion homeostasis (GO:0098771)	+	2.37	1.10E-04	1.97E-02
chemical homeostasis (GO:0048878)	+	2.35	3.85E-05	9.82E-03
homeostatic process (GO:0042592)	+	2.05	3.56E-04	4.24E-02
regulation of biological quality (GO:0065008)	+	1.75	3.83E-04	4.02E-02

intracellular signal transduction (GO:0035556)	+	1.71	1.90E-04	2.83E-02
signal transduction (GO:0007165)	+	1.53	2.44E-05	7.27E-03
cellular response to stimulus (GO:0051716)	+	1.4	3.62E-04	4.05E-02
cellular process (GO:0009987)	+	1.22	7.56E-05	1.50E-02
sensory perception of chemical stimulus (GO:0007606)	-	0.1	1.30E-06	1.17E-03
detection of chemical stimulus involved in sensory perception (GO:0050907)	-	< 0.01	2.33E-08	4.17E-05

Supplementary Notes

Supplementary Note 1: **Comparison of different Hi-C scaffolding programs**

Three different scaffolders, 3D-DNA[1], Proximo (Phase Genomics) and SALSA2[2] were evaluated for building scaffolds using the following parameters.

For 3D-DNA, raw reads were aligned with juicer git commit d940e9e012a75822ff3f5a9ed7b3ecf08999df01 with the options -z `pwd`/reference/asm.fasta -y `pwd`/reference/asm_MboI.txt -q phillippy.q -l phillippy.q -D software/juicer/ -d `pwd` -p `pwd`/reference/chr.sizes. Scaffolding with 3D de novo assembly: version 170123 used the command -m haploid -t 15000 -s 2 -c 30 asm.fasta merged_nodups.txt for both haplotypes.

Phase Genomics' Proximo Hi-C genome scaffolding platform (git commit 145c01be162be85c060c567d576bb4786496c032) was used to create chromosome-scale scaffolds from the contig assembly as described in Bickhart et al[3]. As in the LACHESIS method[4], this process computes a contact frequency matrix from the aligned Hi-C read pairs, normalized by the number of Sau3AI restriction sites (GATC) on each contig, and constructs scaffolds in such a way as to optimize expected contact frequency and other statistical patterns in Hi-C data. Approximately 40,000 separate Proximo runs were performed to optimize the number of scaffolds and scaffold construction in order to make the scaffolds as concordant with the observed Hi-C data as possible.

Details for the SALSA2 run is given in the Methods section of the manuscript.

We evaluated the performance of each scaffolder using a heuristic scoring method involving mapping genetic markers to each set of scaffolds. Each method was scored based on the inverse of the number of scaffolds required to cover the base chromosome (N), the difference in cumulative length of these scaffolds compared to the analog chromosome in ARS-UCD1.2 in megabases (L), and the number of contig order errors within each scaffold. The score function can be summarized by this equation:

$$Score = \frac{1}{(N+i+b+L)} \qquad (1)$$

where “i” represents the number of contig inversions and “b” represents the number of continuity breaks detected in the genetic map based on their positions in ARS-UCD1.2. Scoring was performed on a per-chromosome basis with replacement of scaffolds that had previously mapped to other chromosomes. Using this method, SALSA2 gave most chromosomes with the highest scores and hence was the chosen to use for scaffolding.

Supplementary Note 2: **Comparison of optical map based scaffolding approaches**

De novo optical map assembly and haplotype resolution

Approximately 450 Gb of sequence, representing ~167x coverage, with molecule length >150 kb was aligned to the Brahman and Angus haplotig assemblies respectively to produce haplotype resolved scaffolds of each breed. Alignment of each molecule to the haplotigs of each breed was given a confidence score, which is the log of the alignment p-value. If the confidence score was 2 points higher for one breed haplotigs than the other, then the molecule was binned with the breed with the higher score. Molecule with alignments having almost equal score, defined as within 2 confidence score points of each other, were considered as homozygous and were randomly binned to one of the two breeds to keep the

coverage in homozygous and heterozygous regions uniform. In summary, 135 Gb of the molecules aligned to Angus only, 141 Gb of the molecules aligned to Brahman only, 110 Gb aligned to both Angus and Brahman and were binned evenly. About 64 Gb of molecules aligned to neither the Brahman nor the Angus haplotigs and were also binned randomly to one or the other breed.

For the optical map assembly, a pairwise comparison of all DNA molecules was used to create a layout overlap graph, which was then used to create the initial consensus genome maps. By realigning molecules to the genome maps and using only the best-matched molecules, the label positions on the genome maps were refined and used to validate the minimum assembly tiling path. Next, the software aligned molecules to genome maps and extended the maps based on the molecules aligning beyond the map ends. Overlapping genome maps were then merged. This process was repeated 5 times before a final refinement step was applied to "finish" all genome maps.

To analyse the advantages of haplotype-resolved vs haplotype-unaware optical map construction to guide scaffolding, we generated scaffolds based on the conventional approach that used all Bionano molecules. With about 450 Gbp (molecules >150kbp) of molecules collected from the Brahman-Angus offspring, the *de novo* assembly was 3.37 Gbp with an N50 of 71.11 Mbp. This approach was not biased a priori by assigning parental haplotype and instead resolved haplotype in a *de novo* manner. The advantages of this approach were an increased sequence coverage as molecules were not split to each haplotype and there was no reliance on molecule alignment to haplotigs to bin them to each breed. However, as parental alleles were not separated prior to assembly, switching

between parental alleles in the scaffolds is possible. Furthermore, as the scaffolding algorithm was unaware that the Angus contigs have no X sequences, the final scaffold length for the Angus assembly was longer than expected as the genome map used to guide scaffolding included the X chromosome. The Angus sequence that aligned to the X chromosome genome map likely belonged to the Y chromosome pseudoautosomal region, which is known to have high sequence identity with the X chromosome.

Supplementary Table 8: **Input dataset used to perform optical map-based assemblies using the haplotype-resolved versus the conventional haplotype unaware approach.**

Description	Angus × Brahman Offspring	Angus-Selected	Brahman-Selected
Data collected (molecules > 150 kbp)	480 Gbp	222 Gbp	228 Gbp
Effective coverage of reference	126x	65x	65x
Assembly size	3.37 Gbp	2.79 Gbp	2.87 Gbp
Genome map N50	71.11 Mbp	33.97 Mbp	28.62 Mbp

Haplotype-resolved scaffold assembly

Haplotype-resolved scaffold assembly was performed using the contigs of Brahman and Angus separately, and the Bionano genome map was assembled using standard parameters in Bionano Access (Bionano Solve 3.2.1). For both the Brahman-selected and Angus-selected assemblies, about 98% of the sequences were incorporated into the final hybrid assemblies with N50s of about 34 Mbp. The Angus-selected scaffold has a length of 2.53 Gbp while the

Brahman-selected scaffold has a length of 2.64 Gbp. The N50s of the Angus-selected and Brahman-selected assemblies are limited by the breakage of the Brahman and Angus sequence assemblies in potentially random regions of the genome, which creates a bias in the molecule alignment step during molecule selection. During the scaffolding process, 29 and 36 discrepancies were identified in the Angus and Brahman scaffolds, respectively. These were most likely sequence chimeras, and breaks were introduced in the sequence contigs.

Supplementary Table 9: **Angus-selected Bionano assembly with Angus contigs.**

Statistic	Original Bionano	Original sequence	Sequence used in scaffold	Scaffold	Scaffold + leftover unscaffolded sequence
Number of contigs	597	1747	397	217	1595
N50 (Mbp)	33.97	29.44	32.50	35.49	35.24
Total length (Mbp)	2790.07	2573.81	2512.48 (97.6%)	2526.00	2587.27

Supplementary Table 10: **Brahman-selected Bionano assembly with Brahman contigs.**

Statistic	Original Bionano	Original sequence	Sequence used in scaffold	Scaffold	Scaffold + leftover unscaffolded sequence
Number of contigs	493	1585	421	154	1353
N50 (Mbp)	28.62	23.45	21.99	32.70	31.74
Total length (Mbp)	2867.64	2678.77	2632.15 (98.3%)	2644.13	2690.21

Conventional optical map scaffold assembly

Scaffolding between the Brahman-Angus offspring Bionano map with the Brahman sequence and the Angus sequence were also performed respectively (Bionano Solve 3.2.2). The assemblies have lengths of about 2.65 Gbp with N50 of 84 Mbp. Potential sequence errors (133 in the Angus and 65 in the Brahman) were detected and corrected while running the Bionano scaffolding pipeline (Bionano Solve 3.2.2).

Supplementary Table 11: **Offspring scaffolding with Angus contigs.**

Statistic	Original Bionano	Original sequence	Sequence used in scaffold	Scaffold	Scaffold + leftover unscaffolded sequence
Number of contigs	1026	1747	496	111	1414
N50 (Mbp)	71.11	29.44	29.44	84.07	84.07
Total length (Mbp)	3370.21	2573.81	2518.93 (97.9%)	2643.34	2693.03

Supplementary Table 12: **Offspring scaffolding with Brahman contigs.**

Statistic	Original Bionano	Original sequence	Sequence used in scaffold	Scaffold	Scaffold + leftover unscaffolded sequence
Number of contigs	1026	1585	433	87	1282
N50 (Mbp)	71.11	23.45	21.82	84.31	84.31
Total length (Mbp)	3370.21	2678.77	2632.43 (98.3%)	2660.03	2705.93

Supplementary Note 3: **Genome annotation of UOA_Brahman_1 using the NCBI annotation pipeline**

The NCBI Eukaryotic Genome Annotation Pipeline was used to annotate genes, transcripts, proteins and other genomic features on the Bos indicus haplotype (GCF_003369695.1). The methodology for producing NCBI Bos indicus x Bos taurus Annotation Release 100 (AR 100) was as described for the UMD_CASPUR_WB_2.0 assembly[5]. The evidence aligned to the genome and used for gene prediction was made up of transcript data from the same individual that provided the sample for the genomic sequence: 56,550 PacBio Iso-Seq reads from brain, liver, kidney, placenta, skeletal muscle, lung, and heart, and 1.7 billion RNA-Seq reads from brain, liver, lung, placenta and skeletal muscle. The other evidence used were: 8 billion RNA-Seq reads from 15 *Bos indicus* tissue samples, transcripts and proteins from *Bos*

taurus (14,281 known RefSeq proteins, 19, 584 GenBank proteins, 1,583,270 ESTs), and 52,350 human known RefSeq proteins.

The resulting annotation consists of 20,846 protein-coding genes, 16,398 of which have an ortholog to human. Only 135 protein-coding genes are missing a start or a stop codon and are marked as partial. Where necessary, the annotation pipeline introduced differences between the predicted models and the genomic sequence to compensate for frameshift-causing genomic insertions or deletions that are not supported by protein alignments. These "corrected" proteins are prefixed with 'LOW QUALITY PROTEIN' and should be considered as lower confidence. As an additional testament to the quality of the assembly, only 677 protein coding genes required such a "correction".

Supplementary Table 13: **Comparisons of the number of corrected coding sequences in selected mammalian species.** Although our Brahman reference (UOA_Brahman_1) was not polished with short reads using a pipeline such as Pilon used for ARS-UCD1.2, the number of corrected CDS is very similar. This suggests polishing with PacBio reads alone, without mixing reads representing different haplotypes, has resulted in a consensus assembly with less INDEL errors, which is a common problem that leads to correction of CDS necessary in the annotation step.

Scientific name	Assembly name	Sequencing technology	Number of corrected CDS
Bos taurus	ARS-UCD1.2	PacBio; Illumina NextSeq 500; Illumina HiSeq; Illumina GAII	622
Bos indicus x Bos taurus	UOA_Brahman_1	PacBio Sequel; PacBio RSII; Illumina NextSeq	677
Capra hircus	ARS1	PacBio	946
Ovis aries musimon	Oori1	Not listed	979

Bos indicus	Bos_indicus_1.0	SOLiD	1383
Bison Bison	Bison_UMD1.0	454; Illumina HiSeq	1,448
Ovis aries	Oar_rambouillet_v1.0	HiSeq X Ten; PacBio RS II	1,646
Bubalus bubalis	UOA_WB_1	PacBio	1943
Bos mutus	BosGru_v2.0	Illumina HiSeq; Illumina GA	1954
Ovis aries	Oar_v4.0	Illumina GAII; 454; PacBio RSII	4524

Supplementary Note 4: **Genome annotation of UOA_Angus_1 and UOA_Brahman_1 using the Ensembl annotation pipeline**

Four major classes of evidence were used to create a set of candidate transcripts: pooled high-quality Iso-Seq transcripts from seven tissues (brain, liver, lung, skeletal muscle, placenta, kidney, and heart) of the sequenced F1 hybrid fetus, short read RNA-seq data from five tissues (brain, liver, lung, skeletal muscle, and placenta) of the F1 hybrid fetus, along with publicly available *Bos taurus taurus* and *Bos taurus indicus* data, human transcripts mapped from the GENCODE[6] gene set using pairwise whole genome alignment and finally vertebrate proteins with experimental evidence from UniProt[7] (see below).

Supplementary Table 14: **Initial transcript models for each major input data type for Ensembl annotation.** The unfiltered transcript models came from the initial alignments of each datatype, and later in the annotation process low quality, fragmented and redundant models were removed to produce the finalized gene/transcript models.

Data type	Maternal initial models	Paternal initial models
Iso-Seq (7 pooled tissues)	165822	161968
Short read RNA-seq (32 tissues + 1 merged)	1167059	1150303

Human GENCODE mapping	53178	52242
UniProt known vertebrate proteins	542891	533051

Data from each locus was assessed to remove low quality models and then collapsed into a final gene model with an associated non-redundant transcript set. During the collapsing process, priority was given to transcript models based on the transcriptomic (Iso-Seq and RNA-Seq) data over models derived from homology. For protein coding genes, we also assessed the coverage of the open reading frame (ORF) in relation to known vertebrate proteins. The most complete model was chosen at each locus. In cases where the transcriptomic data appeared fragmented in comparison to the homology data, the homology data were included for completeness. Similarly, for regions where there were no transcriptomic data, we included models based on homology if there was a sufficiently good alignment.

Each gene was then classified as protein coding, long non-coding or pseudogene based on an analysis of the alignment information present at each locus. Genes with transcripts matching known proteins, which did not display multiple structural abnormalities, were classified as protein coding. If a gene matched a known protein but had several problems with the underlying structure (i.e. non-canonical splice sites, abnormally short introns, high level of repeat coverage, no evidence of expression), we classified it as a pseudogene. Single exon genes were assessed for evidence of retrotransposition based on the presence of a multi-exon gene with a highly similar ORF elsewhere in the genome. Such single exon genes were classed as processed pseudogenes. If a gene fell into none of the previous categories, did not overlap a protein coding gene and had been constructed from transcriptomic data, it

was considered as a potential lncRNA. The lncRNA set was filtered to remove transcripts that did not have at least two valid splice sites or cover 1000 bp.

In addition to the above, a small non-coding RNA annotation was produced. The miRNA genes were identified by running a BLAST[8] of miRbase[9] against the genome and then passing the results into RNAfold[10]. Results were post filtered to remove poor quality alignments or alignments that were covered by repeats. For other small non-coding gene types, Rfam[11] was used to scan against the genome and the results were passed into Infernal[12]. Both the maternal and paternal gene sets are available as part of Ensembl release 97. More information on the annotation is available at http://asia.ensembl.org/info/genome/genebuild/2019_06_hybrid_cattle_gene_annotation.pdf

Supplementary Note 5: **Further assembly evaluation**

The completeness of the genome from contig to chromosome-level assembly was assessed using the Benchmarking Universal Single-Copy Orthologs (BUSCO) v2.0.1[13]. The mammalia_odb9 lineage-specific profile that contains 4,104 BUSCO gene groups was tested against the Brahman and Angus genome assemblies using the option -m geno. In addition to testing for completeness in gene space using BUSCO, the assembly base quality values (QV) and other assembly metrics including compression/expansion (CE) errors were calculated using BWA MEM[14], FRCBam[15] and Freebayes[16] as previously described[17].

For consistency in the evaluation of some recently assembled mammalian genomes, we assessed the error rates using the same method described for the water buffalo[18] and goat[3]

genomes. Briefly, short-insert Illumina WGS reads from the parents of the sequenced hybrid F1 animal were aligned to the corresponding haplotype-resolved assemblies using BWA MEM[14]. These short reads were not used in the assembly process and hence they served as an independent dataset to evaluate the genomes. We used the reference-free assembly validation software, FRCBam[15] to generate feature response curves on the Angus and Brahman assemblies in order to identify compression/expansion (CE) errors in the genomes. Putative erroneous bases in each assembly were also identified using FreeBayes[16]. The results are tabulated in Supplementary Table 2. Commands used to generate all assembly quality assessment metrics can be found in the GitHub repository (https://github.com/lloydlow/BrahmanAngusAssemblyScripts).

Supplementary Note 6: **Identification of selective sweep regions**

To uncover genetic variants involved in indicine adaptative selection, we designed a strategy to identify selective sweeps using the haplotype-resolved Brahman genome. This method is analogous to the Cross Population Extended Haplotype Homozygosity (XP-EHH)[19] in that genomic regions are searched for selected alleles that are approaching fixation in the Brahman population but remains polymorphic in six other taurine breeds populations. Only SNPs from the 38 individuals representing seven breeds from the USMARC Beef Diversity Panel version 2.9 (MBCDPv2.9) were considered in the selective sweep analysis.

The selection sweep method

If a genomic region has a high level of homozygosity within Brahman individuals, but the same region contains segregating polymorphic variants in taurine breeds (or visa versa), it can be considered as a candidate region for a selective sweep. An alternative explanation

for such SNP patterns, which cannot be discounted on the available evidence, is genetic drift. Individuals chosen for selective sweep detection were sires with minimal pedigree relationships to ensure sampling of diverse alleles suitable for a population genetics study[20]. The methodology for is similar to other recent studies to identifying selective sweep in cattle[21,22]. The present study had the advantage of using a haplotype-resolved Brahman genome to call SNPs rather than relying on the poorer resolution taurine-based reference, UMD3.1.1.

The proportion of alternate alleles in fixed size windows for the 38 animals representing seven breeds was calculated using the Brahman reference genome. To calculate the proportion of alternate alleles, the annotated genotype data was processed in R using custom scripts to ensure good quality data was included. Only genotypes with complete calls for all animals were retained. For Brahman genotypes, a further requirement of at least five mapped reads was imposed to ensure sufficient coverage to detect the presence of alternate alleles. A genotype labelled as 0/0 is homozygous and the same as the reference and hence there is no alternate allele. The maximum number of alternate alleles possible at a locus for an individual is two (e.g. a genotype call of 1/1).

Let

$n =$ Number of individuals

$c =$ Count of alternate allele at a particular position per individual

$m =$ Number of SNPs in the window

Proportion of alternate allele at position j is

$$\frac{\sum_{i=1}^{n} c_{ij}}{2n} \qquad (2)$$

where c_{11} refers to the count of alternate allele for the 1st individual at the 1st SNP in the window.

Therefore, for each window, mean proportion of alternate allele (MP) is,

$$MP = \frac{\sum_{j=1}^{m}\left(\frac{\sum_{i=1}^{n} c_{ij}}{2n}\right)}{m} \qquad (3)$$

Using this formula, indicine individuals were grouped together to calculate proportion mean of alternate allele, and the same process was repeated for the taurine individuals as a group. The figure below shows the proportion of mean alternate alleles in Brahman and the other six taurine breeds as histograms.

Supplementary Figure 10: **Histograms of the mean proportion alternate allele in Brahman and the other six taurine breeds.** The red line to the left in the Brahman plot indicates the bottom 5% percentile (i.e. extremely low polymorphism). The red line to the right in the taurine plot indicates the top 10% percentile with high polymorphic divergence relative to the Brahman.

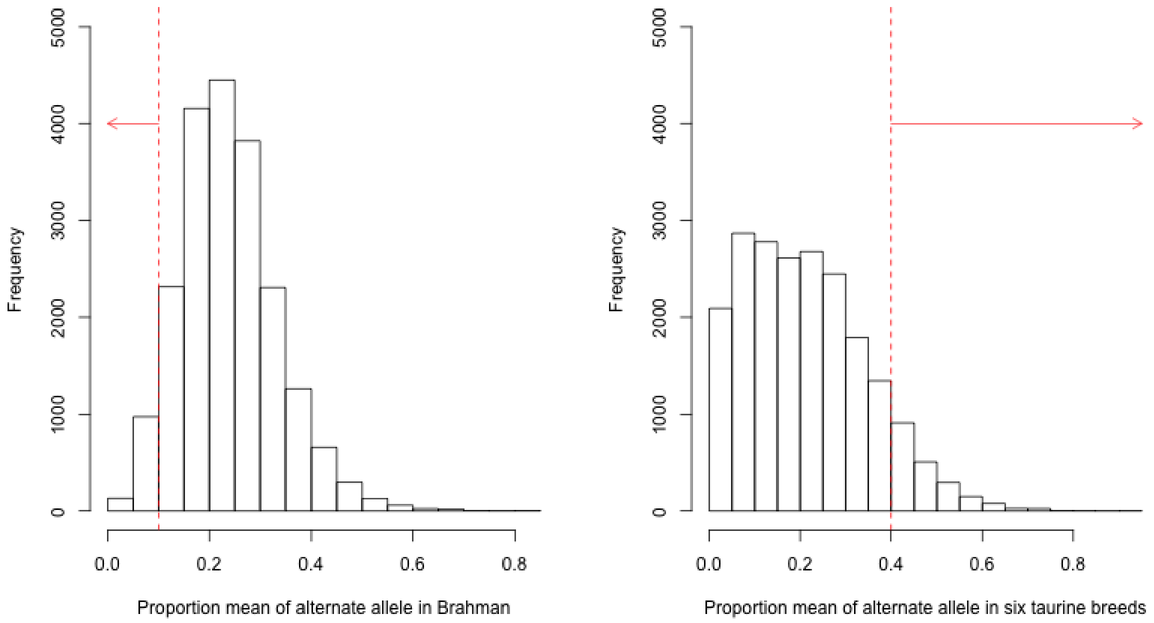

Three fixed size windows (50 kb, 100 kb and 150 kb) were tested to check which would cover sufficient SNPs for the calculation of alternate alleles, and to determine the distance of consecutive SNPs. The table below shows the comparison of different window sizes for the dataset.

Supplementary Table 15: **Comparisons of different window sizes for SNP counts and consecutive SNP distances in each window.**

Window size (kb)	Mean SNP count	Median SNP count	Mean consecutive SNP distance (bp)	Median consecutive SNP distance (bp)	Percentage of windows with at least 10 SNPs
50	8.381	8.000	3341	3306	36%
100	16.39	16.00	5321	5028	81%

150	24.39	24.00	6122	5562	92%

The 50 kb window size was not appropriate for the selective sweeps analysis because many windows contained less than 10 SNPs. When the window size selected was 100 kb, 81% of all windows contained at least 10 SNPs. Additionally, the distance between consecutive SNPs was ~800 bp shorter, on average, when compared to the 150 kb window size. In other words, there was higher density of SNPs when 100 kb windows were chosen. Regardless of whether the 100 kb or the 150 kb window size was chosen, the final gene list in the selection intervals overlapped substantially, although more candidate genes were detected using 100 kb windows. Only windows with at least 10 SNPs were considered for calculation of mean proportion of alternate alleles.

Pathway analysis

Candidate selective sweep genes in the Brahman genome were analyzed for potential genes overrepresentation in biological pathway using PANTHER 14.1[23]. As there are no *Bos taurus indicus* specific biological pathways annotated, the candidate Brahman genes were mapped to the corresponding Hereford ARS-UCD1.2 Ensembl annotation release96 prior to running PANTHER. The selective sweep intervals were also searched against cattle quantitative trait loci (QTL) from Animal QTL database[24].

Interpretation of selective sweep results

We compared the SNP patterns of 5 Brahman with 33 individuals from six taurine breeds to identify signatures of selective sweeps (Supplementary Note 6 Figure 2a). We searched 100 kb windows spanning the whole genome for those where there was high level of

homozygosity within Brahman individuals but with more segregating polymorphic variants in taurine breeds. This identified a total of 128 genes in 60 selective sweep intervals. Among these candidate selected genes, 80% were protein-coding, 1 was a pseudogene and the remainder were RNA-based genes (Supplementary Table 5). No biological pathways were found to be significantly over-represented among the positively selected protein-coding genes. The heat shock protein HSPA4*,* a member of the Hsp70 family, was amongst genes identified as under selection (Supplementary Note 6 Figure 2b). This region was also found in a search for selective sweeps in African cattle[22], which we would not expect by chance. We also identified *DNAJC13*, a member of a gene family known to act as co-chaperones of heat-shock proteins, in another selective sweep region.

A total of 231 unique QTL covering all six major QTL types listed in the cattle QTL database overlapped with positively selected genomic regions. The major QTL types were reproduction (26%) followed by exterior phenotype (22.9%) and milk (22.1%) traits (Supplementary Note 6 Figure 2c). Ten of 60 selective sweep intervals did not overlap with any of the currently identified QTL.

Supplementary Figure 11: **Selective sweep analysis in Brahman.** a) An overview of the strategy used to identify selective sweep regions by aligning and calling SNPs in 100-kb windows on the Brahman reference. The variant under selection is highlighted in yellow. b) An example of genotype calls for *HSPA4*, a gene residing in one of the selective sweep intervals, using partial results from Angus and Brahman individuals. Genotype shown as "." means it follows the Brahman reference, which is haploid, and "an" denotes Angus whereas "br" denotes Brahman. The position indicates adjusted position starting from 66,130,388 bp in

UOA_Brahman_1, chromosome 7. c) Overlap of selective sweep intervals with cattle QTL categorized by types. Only unique QTL IDs were used.

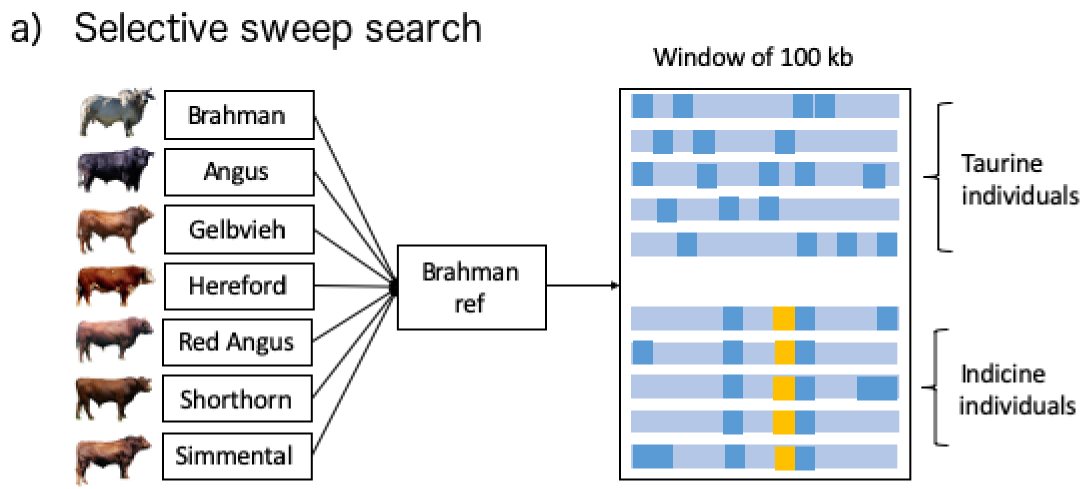

b) HSPA4

Position	1	18435	18436	18437	22423	29515	36924	36948	47185	67749
Brahman ref	A	C	A	C	C	A	T	T	G	G
br1	GG	..	..	..	..	..	..	..	..	..
br2	..	..	..	..	..	..	..	..	..	..
br3	..	..	..	..	..	..	..	..	..	..
br4	GG	..	..	..	..	..	..	..	..	.A
br5	.G	.T	.T	.T	..	..	..	.C	..	..
an1	GG	..	..	..	..	.G	.A	CC	..	..
an2	GG	.T	.T	.T	..	.G	.A	CC	..	..
an3	GG	TT	TT	TT	.T	..	..	CC	..	..
an4	GG	.T	.T	.T	..	.G	.A	CC	..	..
an5	GG	TT	TT	TT	..	..	..	CC	..	..
an6	GG	TT	TT	TT	TT	..	..	CC	..	..

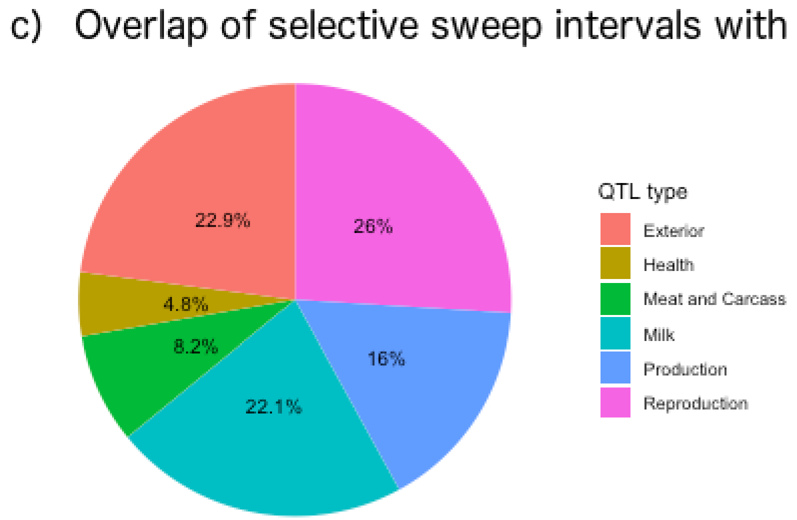

The significant differences in phenotype, energy metabolism and adaptation to heat stress of indicine cattle have been linked to the thyroid hormone axis[25–27]. One of the selective sweep regions in Brahman contains thyroid hormone receptor β (THRB), a ligand-activated pleiotropic transcription factor that modulates the expression of a large number of genes[28]. Thyroid hormones are intrinsically connected to the growth hormone - insulin-like growth factor axis (GH-IGF)[29]. Insulin-like growth factor 1 receptor (IGF1R) was found in another selective sweep region. Polymorphisms in *IGF1R* have been associated with age of puberty in Brahman cattle[30]. In comparison with taurine cattle Brahman tend to reach puberty late, which may have been under positive selection as a consequence of adaption to harsh tropical environments, ensuring that cows are more mature and robust at the time of first calving.

Quantitative trait loci for reproduction featured prominently in the comparison of Brahman selective sweep regions with known cattle QTL. Amongst the reproduction traits, QTL related to calf size and calving ease were overrepresented. Brahman cows deliver a small calf that is less likely to result in dystocia and still birth[31], which is one major benefit of the introgression of indicine genetics into more productive taurine breeds. Selective sweep regions thus provide candidate genes for maternal control of birth weight.

Supplementary Note 7: **Further Iso-Seq analysis**

After exclusion of SNPs with less than 40-fold Iso-Seq read coverage and those in non-transcribed regions, IsoPhase identified 5806 genes with 52,270 phased transcripts.

SNP calls missed by IsoPhase are either in homopolymer regions or have low coverage

There are 8,093 (substitution) SNP calls that are missed by IsoPhase but called jointly by RNA-Seq and genomic data. 5589 (69%) of the missed calls are either within or adjacent to a homopolymer (HP) region. IsoPhase defines a HP region as a stretch of 4 consecutive identical bases and will not call a SNP if it is inside a HP or immediately adjacent to a HP region.

Of the remaining 1813 missed calls (22%), have effective base coverage less than 40 in the Iso-Seq data. Effective base coverage is different from full-length Iso-Seq read coverage because after alternative splicing, certain exons, introns, or UTRs may have lower coverage despite meeting the initial 40 fold threshold requirement to run IsoPhase.

Of the missed PacBio calls, 91% are either in or adjacent to HP regions, or have insufficient base coverage. Manual inspection of the remaining 9% of the missed calls suggest that either they were also adjacent (but not immediately) to HP regions, or had low coverage resulting in insignificant P-values to pass the IsoPhase SNP call threshold.

IsoPhase-unique SNP calls are dominantly A to G calls, which suggests RNA editing

There are 2830 SNP calls unique to IsoPhase, 1776 SNP calls unique to RNA-seq, and 2651 SNP calls unique to genome. We tally the unique calls by Reference to Alternative SNP call in the figure below using the transcribed orientation as the sense strand and show that A to G is the most dominant unique call in IsoPhase and RNA-Seq data. It is not clear why PacBio Iso-Seq is the only platform to call certain SNPs.

Supplementary Figure 12: **Percentages of SNP type across those unique in each of PacBio Iso-Seq, RNA-Seq and genome WGS datasets.**

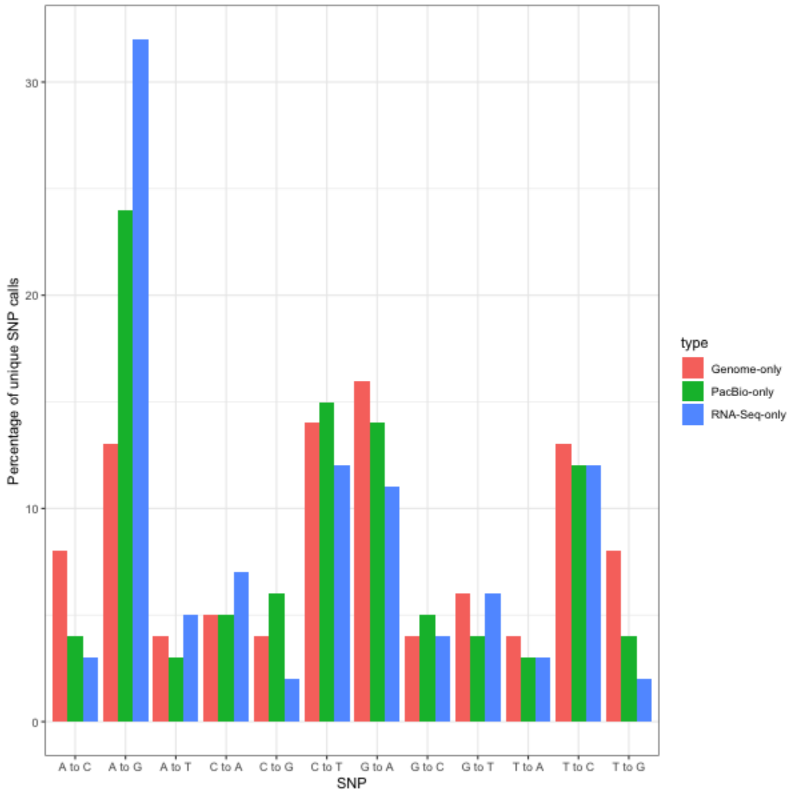

Rarefaction analysis of covered genes and transcripts

The subsampling of full-length non-concatamer (FLNC) reads at the level of genes and transcripts showed that brain, kidney and heart datasets were reaching a plateau, suggesting sequencing depth was adequate to discover the majority of transcripts for the annotation process. To ensure saturation of transcripts, additional data was produced for the brain sample, which was run with seven SMRT cells. By extrapolating from the rarefaction curve, 10 SMRT cells per tissue to gather ~1 million FLNC should ensure saturation of transcripts at this developmental stage.

Supplementary Figure 13: **Rarefaction analysis of seven tissues in F_1 animal at the level of a) gene, b) transcript.**

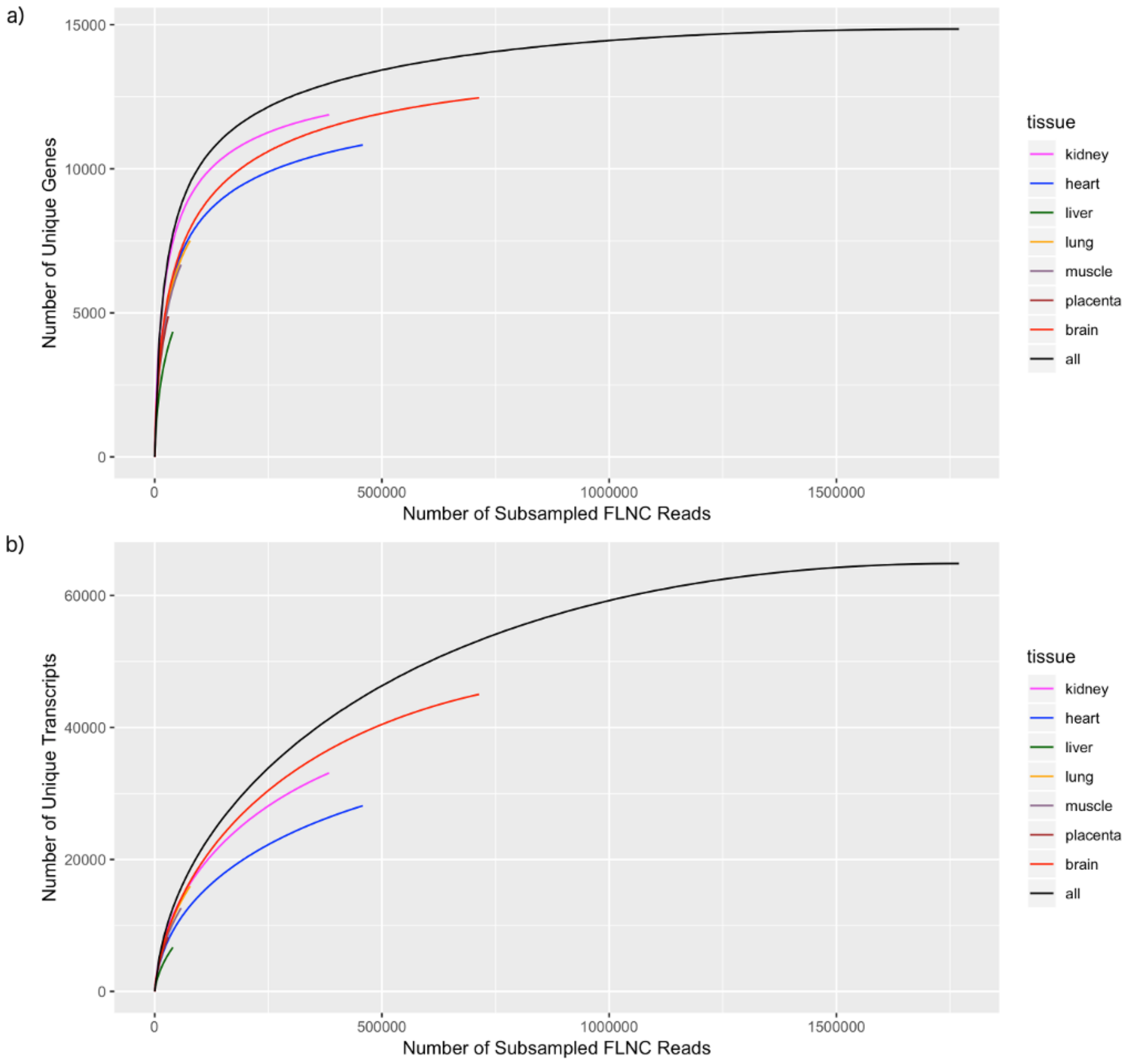

Supplementary References

1. Dudchenko, O. *et al.* De novo assembly of the Aedes aegypti genome using Hi-C yields chromosome-length scaffolds. *Science* **356**, 92–95 (2017).

2. Ghurye, J. *et al.* Integrating Hi-C links with assembly graphs for chromosome-scale assembly. *bioRxiv* 261149 (2019). doi:10.1101/261149

3. Bickhart, D. M. *et al.* Single-molecule sequencing and chromatin conformation

capture enable de novo reference assembly of the domestic goat genome. *Nat. Genet.* **49**, 643–650 (2017).

4. Burton, J. N. *et al.* Chromosome-scale scaffolding of de novo genome assemblies based on chromatin interactions. *Nat. Biotechnol.* **31**, 1119–1125 (2013).

5. Williams, J. L. *et al.* Genome assembly and transcriptome resource for river buffalo, Bubalus bubalis (2n = 50). *Gigascience* **6**, 1–6 (2017).

6. Frankish, A. *et al.* GENCODE reference annotation for the human and mouse genomes. *Nucleic Acids Res.* **47**, D766–D773 (2019).

7. UniProt: a worldwide hub of protein knowledge. *Nucleic Acids Res.* **47**, D506–D515 (2019).

8. Altschul, S. F., Gish, W., Miller, W., Myers, E. W. & Lipman, D. J. Basic local alignment search tool. *J. Mol. Biol.* **215**, 403–10 (1990).

9. Kozomara, A., Birgaoanu, M. & Griffiths-Jones, S. miRBase: from microRNA sequences to function. *Nucleic Acids Res.* **47**, D155–D162 (2019).

10. Gruber, A. R., Lorenz, R., Bernhart, S. H., Neuböck, R. & Hofacker, I. L. The Vienna RNA websuite. *Nucleic Acids Res.* **36**, W70-4 (2008).

11. Kalvari, I. *et al.* Rfam 13.0: shifting to a genome-centric resource for non-coding RNA families. *Nucleic Acids Res.* **46**, D335–D342 (2018).

12. Nawrocki, E. P. & Eddy, S. R. Infernal 1.1: 100-fold faster RNA homology searches. *Bioinformatics* **29**, 2933–5 (2013).

13. Simão, F. A., Waterhouse, R. M., Ioannidis, P., Kriventseva, E. V & Zdobnov, E. M. BUSCO: assessing genome assembly and annotation completeness with single-copy orthologs. *Bioinformatics* **31**, 3210–2 (2015).

14. Li, H. & Durbin, R. Fast and accurate short read alignment with Burrows--Wheeler

transform. *Bioinformatics* **25**, 1754–1760 (2009).

15. Vezzi, F., Narzisi, G. & Mishra, B. Reevaluating Assembly Evaluations with Feature Response Curves: GAGE and Assemblathons. *PLoS One* **7**, e52210 (2012).

16. Garrison, E. & Marth, G. Haplotype-based variant detection from short-read sequencing. *arXiv:1207.3907* (2012).

17. Koren, S. *et al.* De novo assembly of haplotype-resolved genomes with trio binning. *Nat. Biotechnol.* **36**, 1174–1182 (2018).

18. Low, W. Y. *et al.* Chromosome-level assembly of the water buffalo genome surpasses human and goat genomes in sequence contiguity. *Nat. Commun.* **10**, 260 (2019).

19. Sabeti, P. C. *et al.* Genome-wide detection and characterization of positive selection in human populations. *Nature* **449**, 913–918 (2007).

20. Heaton, M. P. *et al.* Using diverse U.S. beef cattle genomes to identify missense mutations in EPAS1, a gene associated with high-altitude pulmonary hypertension. *F1000Research* **5**, 2003 (2016).

21. Koufariotis, L. *et al.* Sequencing the mosaic genome of Brahman cattle identifies historic and recent introgression including polled. *Sci. Rep.* **8**, 17761 (2018).

22. Kim, J. *et al.* The genome landscape of indigenous African cattle. *Genome Biol.* **18**, 34 (2017).

23. Thomas, P. D. *et al.* PANTHER: a library of protein families and subfamilies indexed by function. *Genome Res.* **13**, 2129–41 (2003).

24. Hu, Z.-L., Park, C. A., Wu, X.-L. & Reecy, J. M. Animal QTLdb: an improved database tool for livestock animal QTL/association data dissemination in the post-genome era. *Nucleic Acids Res.* **41**, D871-9 (2013).

25. Cowley, J. J., Gutierrez, J. H., Warnick, A. C., Hentges, J. F. & Feaster, J. P. Comparison

of Thyroid Hormone Levels in Hereford and Brahman Cattle. *J. Anim. Sci.* **32**, 981–983 (1971).

26. Obeidat, B. S. *et al.* Metabolic characteristics of multiparous Angus and Brahman cows grazing in the Chihuahuan Desert. *J. Anim. Sci.* **80**, 2223–33 (2002).

27. Façanha, D. A. E. *et al.* The dynamic adaptation of Brazilian Brahman bulls. *J. Therm. Biol.* **81**, 128–136 (2019).

28. Ortiga-Carvalho, T. M., Sidhaye, A. R. & Wondisford, F. E. Thyroid hormone receptors and resistance to thyroid hormone disorders. *Nat. Rev. Endocrinol.* **10**, 582–91 (2014).

29. Forhead, A. J. & Fowden, A. L. Thyroid hormones in fetal growth and prepartum maturation. *J. Endocrinol.* **221**, R87–R103 (2014).

30. Fortes, M. R. S., Li, Y., Collis, E., Zhang, Y. & Hawken, R. J. The IGF1 pathway genes and their association with age of puberty in cattle. *Anim. Genet.* **44**, 91–95 (2013).

31. Comerford, J. W., Bertrand, J. K., Benyshek, L. L. & Johnson, M. H. Reproductive rates, birth weight, calving ease and 24-h calf survival in a four-breed diallel among Simmental, Limousin, Polled Hereford and Brahman beef cattle. *J. Anim. Sci.* **64**, 65–76 (1987).
