# Supplementary material for: Haplotype-resolved genomes provide insights into structural variation and gene content in Angus and Brahman cattle
Source: Nat Commun. 2020 Apr 29;11:2071. doi: 10.1038/s41467-020-15848-y (PMC7190621; doi:10.1038/s41467-020-15848-y)
Supplement: Supplementary file 2 — Reporting Summary [file 41467_2020_15848_MOESM2_ESM.pdf]

nature research

Corresponding author(s): John L. Williams, Timothy P.L. Smith, Stefan Hiendleder

Last updated by author(s): 23/3/20

Reporting Summary

Nature Research wishes to improve the reproducibility of the work that we publish. This form provides structure for consistency and transparency in reporting. For further information on Nature Research policies, see Authors & Referees and the Editorial Policy Checklist .

Statistics

For all statistical analyses, confirm that the following items are present in the figure legend, table legend, main text, or Methods section.

n/a	Confirmed	
☐	☒	The exact sample size (*n*) for each experimental group/condition, given as a discrete number and unit of measurement
☐	☒	A statement on whether measurements were taken from distinct samples or whether the same sample was measured repeatedly
☐	☒	The statistical test(s) used AND whether they are one- or two-sided<br>*Only common tests should be described solely by name; describe more complex techniques in the Methods section.*
☒	☐	A description of all covariates tested
☒	☐	A description of any assumptions or corrections, such as tests of normality and adjustment for multiple comparisons
☒	☐	A full description of the statistical parameters including central tendency (e.g. means) or other basic estimates (e.g. regression coefficient) AND variation (e.g. standard deviation) or associated estimates of uncertainty (e.g. confidence intervals)
☐	☒	For null hypothesis testing, the test statistic (e.g. *F*, *t*, *r*) with confidence intervals, effect sizes, degrees of freedom and *P* value noted<br>*Give P values as exact values whenever suitable.*
☒	☐	For Bayesian analysis, information on the choice of priors and Markov chain Monte Carlo settings
☒	☐	For hierarchical and complex designs, identification of the appropriate level for tests and full reporting of outcomes
☒	☐	Estimates of effect sizes (e.g. Cohen's *d*, Pearson's *r*), indicating how they were calculated

Our web collection on statistics for biologists contains articles on many of the points above.

Software and code

Policy information about availability of computer code

Data collection	Standard code from sequencing providers (PacBio, Illumina) was used to gather sequencing data.
Data analysis	https://github.com/lloydlow/BrahmanAngusAssemblyScripts<br>https://github.com/skoren/ArrowGrid<br>https://github.com/lh3/seqtk<br>http://www.repeatmasker.org

For manuscripts utilizing custom algorithms or software that are central to the research but not yet described in published literature, software must be made available to editors/reviewers. We strongly encourage code deposition in a community repository (e.g. GitHub). See the Nature Research guidelines for submitting code & software for further information.

Data

Policy information about availability of data

All manuscripts must include a data availability statement. This statement should provide the following information, where applicable:

- Accession codes, unique identifiers, or web links for publicly available datasets
- A list of figures that have associated raw data
- A description of any restrictions on data availability

The PacBio reads, Hi-C reads, RNA-Seq, Iso-Seq and Illumina paired-end reads are available in the SRA under BioProject PRJNA432857. The 38 individuals from seven breeds used for variant calls were downloaded from the BioProject PRJNA324822. The assemblies ARS-UCD1.2 (GCF_002263795.1), Bos_taurus_UMD_3.1.1 (GCF_000003055.6), ARS1 (GCF_001704415.1) and UOA_WB_1 (GCF_003121395.1) were downloaded from the NCBI. Intermediary assembly FASTA files and other miscellaneous information are available from the corresponding authors upon request. Annotation files of UOA_Angus_1 and UOA_Brahman_1 are available through Ensembl.

Field-specific reporting

Please select the one below that is the best fit for your research. If you are not sure, read the appropriate sections before making your selection.

- [x] Life sciences
- [ ] Behavioural & social sciences
- [ ] Ecological, evolutionary & environmental sciences

For a reference copy of the document with all sections, see nature.com/documents/nr-reporting-summary-flat.pdf

Life sciences study design

All studies must disclose on these points even when the disclosure is negative.

Sample size	A male F1 hybrid cattle from Angus (sire) crossed to Brahman (dam) was chosen for sequencing. Since we used only a single animal, n = 1. For CNV analysis, we have chosen 38 animals for analysis. The individuals selected for the panel were bulls with minimal pedigree relationships to maximize sampling of diverse alleles suitable for population genetics studies. There were six taurine breeds chosen on the basis that they were unlikely to carry Bos indicus genetics given their history.
Data exclusions	No data exclusion.
Replication	The number of individuals per breed was as follow: six Angus, five Brahman, six Gelbvieh, six Hereford, five Red Angus, five Shorthorn and five Simmental. The number of replicates is based on the maximum number of individuals we can obtain from a resource known as the USMARC Beef Diversity Panel version 2.9.
Randomization	None.
Blinding	None.

Reporting for specific materials, systems and methods

We require information from authors about some types of materials, experimental systems and methods used in many studies. Here, indicate whether each material, system or method listed is relevant to your study. If you are not sure if a list item applies to your research, read the appropriate section before selecting a response.

Materials & experimental systems

n/a	Involved in the study	
x		Antibodies
x		Eukaryotic cell lines
x		Palaeontology
	x	Animals and other organisms
x		Human research participants
x		Clinical data

Methods

n/a	Involved in the study	
x		ChIP-seq
x		Flow cytometry
x		MRI-based neuroimaging

Animals and other organisms

Policy information about studies involving animals; ARRIVE guidelines recommended for reporting animal research

Laboratory animals	All animal work was approved by the Animal Ethics Committee of the University of Adelaide (No. S-094-2005). A Bos taurus indicus female (Brahman) was inseminated with semen from a Bos taurus taurus (Angus) bull. The indicus maternal genetic background of the Brahman dam was confirmed by mitochondrial DNA haplotype analysis. At day 153 post-insemination, dam and conceptus were ethically sacrificed and fetal brain, heart muscle, kidney, liver, lung, skeletal muscle and placenta (cotyledon) tissue were snap frozen in liquid nitrogen and stored at -80°C until further use. All animal work was approved by the Animal Ethics Committee of the University of Adelaide (No. S-094-2005).
Wild animals	Not relevant.
Field-collected samples	Not field collected.
Ethics oversight	Animal Ethics Committee of the University of Adelaide (No. S-094-2005).

Note that full information on the approval of the study protocol must also be provided in the manuscript.
